# New Quinazolin-4(3*H*)-one Derivatives as Potential Antitumoral Compounds: Synthesis, In Vitro Cytotoxicity Against the HepG2 Cell Line, and In Silico VEGFR-2 Targeting-Based Studies

**DOI:** 10.3390/molecules30244719

**Published:** 2025-12-09

**Authors:** Raluca Pele, Gabriel Marc, Brîndușa Tiperciuc, Ioana Ionuț, Anca Stana, Cristina Moldovan, Corina Tatomir, Oana Maria Dragostin, Adrian Pîrnău, Laurian Vlase, Daniel Ungureanu, Ovidiu Oniga

**Affiliations:** 1 Department of Pharmaceutical Chemistry, Faculty of Pharmacy, “Iuliu Hațieganu” University of Medicine and Pharmacy, 41 Victor Babeș Street, 400012 Cluj-Napoca, Romania; raluca.pele@umfcluj.ro (R.P.);; 2 Department of Organic Chemistry, Faculty of Pharmacy, “Iuliu Hațieganu” University of Medicine and Pharmacy, 41 Victor Babeș Street, 400012 Cluj-Napoca, Romania; 3 Department of Radiobiology and Tumor Biology, “Prof. Dr. Ion Chiricuță” Oncology Institute, 34-36 Republicii Street, 400015 Cluj-Napoca, Romania; 4 Research Centre in the Medical-Pharmaceutical Field, Faculty of Medicine and Pharmacy, “Dunărea de Jos” University of Galați, 47 Domneasca Street, 800008 Galați, Romania; 5 National Institute for Research and Development of Isotopic and Molecular Technologies, 67-103 Donath Street, 400293 Cluj-Napoca, Romania; 6 Department of Pharmaceutical Technology and Biopharmaceutics, “Iuliu Hațieganu” University of Medicine and Pharmacy, 41 Victor Babeș Street, 400012 Cluj-Napoca, Romania

**Keywords:** quinazolin-4(*3H*)-one, HepG2 cell line, sorafenib, molecular docking, VEGFR-2

## Abstract

Three new 2,3-disubstituted quinazolin-4(3*H*)-one derivatives (**5a**–**c**) were synthesized by the nucleophilic *S*-alkylation of 2-mercaptoquinazolin-4(3*H*)-one derivatives (**3a**–**c**) with 5-(2-bromoacetyl)-2-hydroxybenzamide (**4**) in alkaline conditions. The final compounds were characterized by recording the IR, MS, ^1^H-NMR, and ^13^C-NMR spectra. The new synthesized compounds **5a**–**c** were evaluated in vitro for their cytotoxic activity using one normal cell line, human foreskin fibroblasts (BJ), and one cancerous cell line, derived from human hepatocellular carcinoma (HepG2). Sorafenib was used as a reference. The obtained results from the in vitro examination suggested that compound **5a** had lower cytotoxic effects on the BJ cells than the positive standard, and compound **5b** exhibited the strongest cytotoxic potential against the HepG2 cell line, being less effective compared to sorafenib. In order to evaluate their pharmacological profiles, the compounds were assessed in vitro and in silico by lipophilicity studies, targeted VEGFR-2 molecular docking, molecular dynamics, and MM-PBSA studies. Additionally, the electronic properties were evaluated by an in silico study of the HOMO and LUMO parameters. Compound **5b** exhibited the most interesting pharmacological profile in comparison with the other compounds due to its bulkier substituent from position 3 of the quinazolinone core.

## 1. Introduction

Hepatocellular carcinoma (HCC) is the most predominant cancer subtype among all liver cancers, often diagnosed in an advanced stage of the disease [1]. Numerous risk factors contribute to the exacerbation of liver cancer, the most common of them being cirrhosis; hepatitis B and C; overweight or obesity; type 2 diabetes, often linked to metabolic-dysfunction-associated steatotic liver disease (MASLD); or exposure to aflatoxins. HCC is an example of a hypervascular tumor; thus, angiogenesis plays a crucial role in its progression [1,2,3].

Receptor tyrosine kinases (RTKs) are key regulators of membrane-bound receptors that control cellular functions such as proliferation and survival. The expression of RTK is typically upregulated in many types of cancer (non-small-cell lung cancer, colorectal cancer, gastrointestinal cancer, thyroid cancer, renal cell carcinoma, etc.).

One of the main RTK families is the vascular endothelial growth factor receptors (VEGFR) family, composed of VEGFR-1, VEGFR-2, and VEGFR-3 isoforms [4]. Vascular endothelial growth factor (VEGF) is a proangiogenic factor involved in HCC, and its overexpression in tumor tissues can be a useful prognostic factor after treatment, in patients with HCC. The latest reported evidence suggests that VEGF/VEGFR-2 signaling pathways are involved in the proliferation, migration, survival, and permeability of vascular endothelial cells.

**Figure 1:**
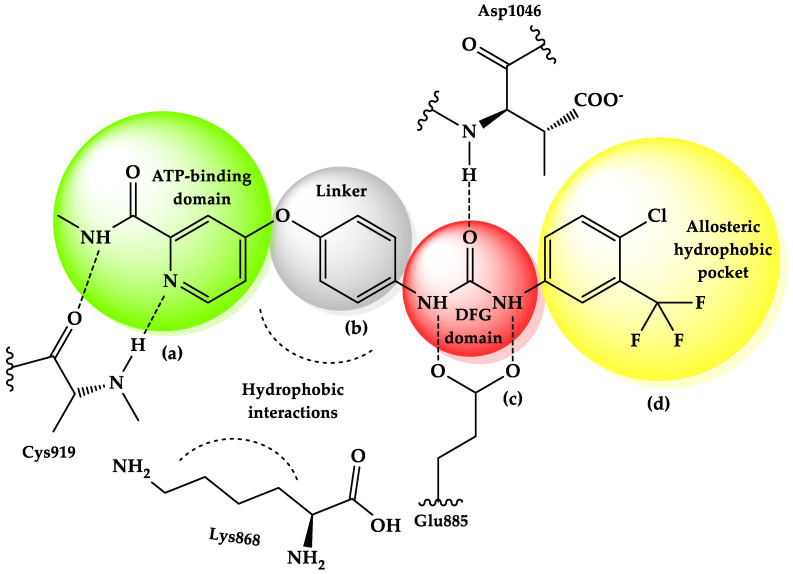
**Sorafenib**—representation of the active site of VEGFR-2 (a = “head” group; b = linker; c = DFG domain; d = tail segment) [4,5,6].

The design and development of new anti-HCC medication can lead to anti-angiogenesis effects through attractive targets, such as the blockade of the VEGF/VEGFR-2 pathway [3]. Sorafenib, a small molecule, is a well-known VEGFR-2 inhibitor approved by the U.S. Food and Drug Administration (FDA) for HCC chemotherapy. It is composed of some common pharmacophoric fragments that interact with VEGFR-2 (Figure 1): (a) a hinge-binding “head” group, represented by an aromatic heterocycle containing a N atom—which fits into the adenine region in the ATP-binding pocket and forms hydrogen bonds—at least one H-bond acceptor (HBA), and/or a H-bond donor (HBD), with Cys919; (b) a “linker”, which spans the gatekeeper residue, occupying the space between the ATP-binding domain and the DFG domain. The linker used here contains an ether-phenyl moiety, where the phenyl interacts with the hydrophobic site (Lys868) in the hinge region; (c) a pharmacophore, which is described as a urea moiety, forming a H-bond acceptor through the C=O group with Asp1046 in the conserved DFG (Asp-Phe-Gly) motif, and two H-bond donors through NH groups with Glu885 of the αC helix (these interactions stabilize the inactive DFG-out conformation of the kinase, which is necessary for the inhibitor drug to bind); (d) a “tail” segment, usually comprising a hydrophobic group, that occupies the allosteric hydrophobic back pocket displayed by the DFG-out conformation [4,5,6]. However, the clinical application of sorafenib is limited because of its side effects during treatment administration, such as anorexia, diarrhea, or heart attack. Moreover, this compound has an unfavorable pharmacokinetic profile, characterized by a low level of solubility and permeability, which increases cytotoxicity and the development of resistance to the drug treatment in cancer cells [7].

Today, in silico studies that can predict the drug-likeness properties are essential for drug discovery and development, determining the safety, efficacy, and overall behavior of potential drug candidates in humans. Drug-likeness, as a concept, involves Lipinski’s rule of five and other additional descriptors, such as solubility, permeability, and stability, that predict the potential of a candidate to become a successful drug [8,9,10,11,12].

**Figure 2:**
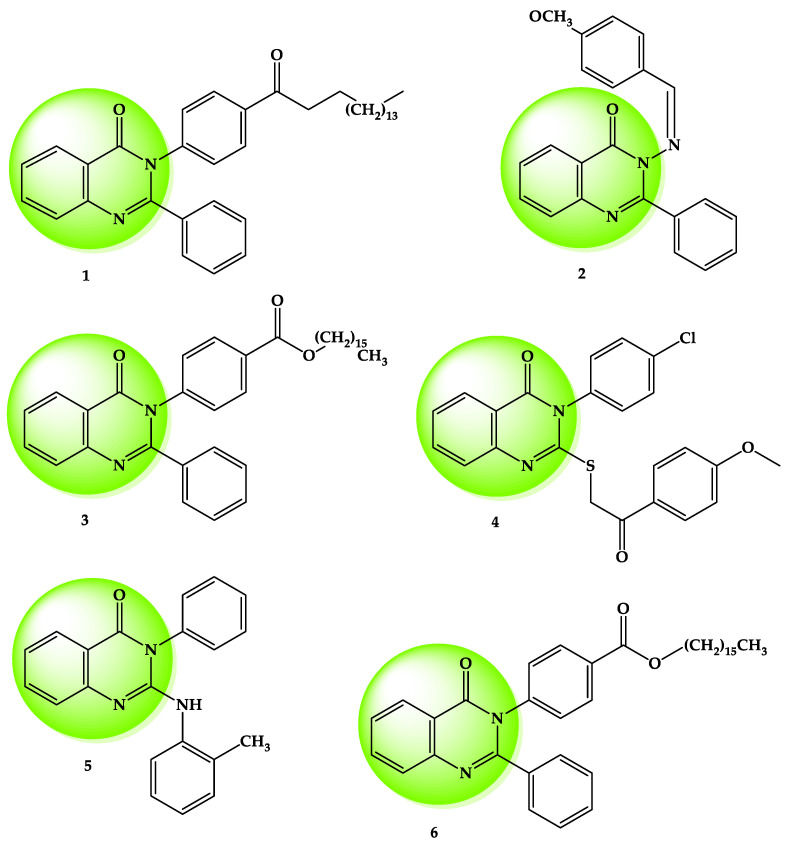
Biologically active compounds with the 2,3-disubstituted quinazolin-4(3*H*)-one scaffold reported in the literature [16,17,18,22,23].

*N*-heterocyclic systems, such as quinolines, quinazolines, quinazolinones, and quinazolindione, represent important scaffolds in the design of antitumor drugs [13,14,15,16,17,18,19,20,21], as well as other heterocycles reported in the literature [8,9]. Small molecules with a 2,3-disubstituted quinazolinone heterocycle structure were studied in various cancer cell lines, including the human hepatocellular carcinoma cell line (HepG2). This heterocycle is widely studied in medicinal chemistry research due to a variety of substituents containing different functional groups that can improve its biological activities (Figure 2) [16,17,18,22,23].

**Figure 3:**
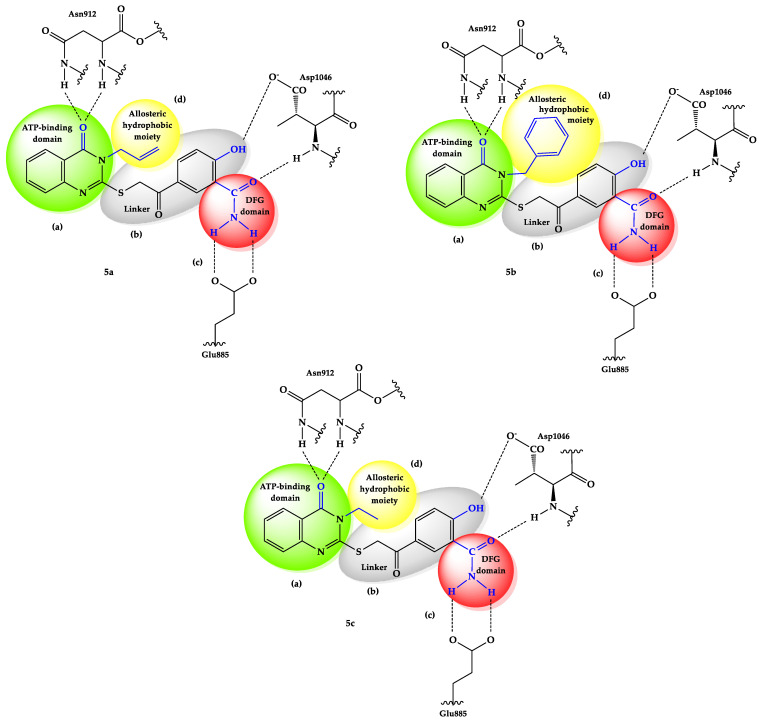
Computational design of **5a**–**c** compounds. (a = “head” group; b = linker; c = DFG domain; d = tail segment) [4,5,6].

Our hypothesis was derived from structure–activity relationship (SAR) studies on sorafenib, which indicated the existence of 2–3 essential regions that interacted with VEGFR-2. Based on these observations, we conducted a preliminary molecular docking study and selected three quinazolinone derivatives with different substituents in the third position. The hetero-aromatic moiety is crucial to enhancing affinity towards the ATP-binding domain [24]. A substitution of the third position from the quinazolinone core was made with some variation in the flexibility of fragments, like allyl, benzyl, or ethyl. We assumed that through higher flexibility, the compounds can be better fixed to the ATP-binding domain and DFG motif, moving away from the distal hydrophobic pocket during the conformational change of DFG-in to DFG-out. Another modification consisted of extending the linker between the ATP-binding domain and DFG motif by changing the *O*-phenyl with a thioether, *S*-acetyl-phenyl spacer. Furthermore, the phenyl ring spacer should enable hydrophobic interactions with the surrounding amino acids from the gate area. The linker connects the quinazoline core to other functional groups, significantly impacting the drug’s binding, solubility, and metabolism [25]. The substitution of the phenyl with a hydroxyl group should increase the capacity to bind to the key amino acids at the active site of VEGFR-2, Glu885 and Asp1046, as well as their hydrophobic interactions with the hydrophobic pockets of the receptor. Also, it was noted to improve the antiangiogenic activity of the reported compounds (Figure 3) [4,5,6,26]. The last modification was represented by the replacement of the urea motif from sorafenib with the more stable carbamide group. Amide or urea groups are crucial linkers on many VEGFR inhibitor drugs that interact with the DFG motif. These groups typically can form two key hydrogen bonds with the backbone of the kinase domain. One hydrogen bond can be with the backbone NH of the aspartate residue in the DFG motif itself (e.g., Asp1046 in VEGFR-2), and the other can be with Glu885, another crucial amino acid localized in the *C*-helix. These interactions stabilize the inactive DFG-out conformation of the kinase, which is necessary for the inhibitor drug to bind.

The desired compounds **5a**–**c** were synthesized and subjected to a series of in silico studies to validate the proposed mechanism of action and to evaluate the drug-likeness properties. Furthermore, in vitro studies were conducted on the cytotoxicity of the compounds on a healthy human foreskin fibroblast cell line (BJ) and on a human hepatocellular carcinoma cell line (HepG2), and the lipophilicity of compounds **5a**–**c** was evaluated.

## 2. Results and Discussion

### 2.1. Chemical Synthesis

After a preliminary molecular docking study, chemical synthesis of our compounds was performed. Based on the obtained binding energies, we synthesized three compounds, **5a**–**c**, with different structural profiles.

**Scheme 1:**
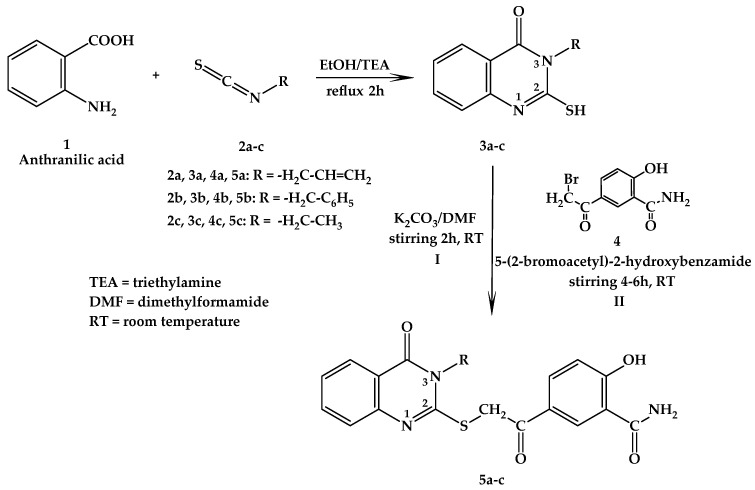
Chemical synthesis of the **5a−c** compounds.

The first step of the synthesis was represented by the reaction of anthranilic acid (**1**) through a nucleophilic addition with three different isothiocyanates (**2a**–**c**)—in alkaline conditions achieved using triethylamine (TEA) in refluxing ethanol—followed by a cyclization reaction, in order to obtain the intermediate 4(*3H*)-quinazolinone compounds (**3a**–**c**) [27,28]. Compounds **5a**–**c** were synthesized via nucleophilic *S*-alkylation of 3-allyl-2-mercaptoquinazolin-4(*3H*)-one (**3a**), 3-benzyl-2-mercaptoquinazolin-4(3*H*)-one (**3b**), or 3-ethyl-2-mercaptoquinazolin-4(*3H*)-one (**3c**) with 5-(2-bromoacetyl)-2-hydroxybenzamide (**4**), with considerable yields, by stirring at room temperature (RT), in alkaline conditions achieved by adding K_2_CO_3_ in DMF (Scheme 1).

### 2.2. Molecular Docking

We started our study with a preliminary screening through in silico molecular docking on VEGFR-2. Because molecular docking is prone to algorithm-specific biases, reporting convergent trends across two distinct engines strengthens confidence in the predicted binding modes. In our study, despite numerical differences, both methods identified similar binding orientations and consistent relative ranking among compounds. This consistency supports the reliability of the findings and reduces the risk that the conclusions depend on a single docking protocol.

The binding affinities of compounds **5a**–**c** and sorafenib to the ATP-binding site of VEGFR-2 are presented in Table 1. The re-docking of sorafenib to the targeted site resulted in a superposable conformation with the crystallized sorafenib, with the coordinates of the heavy atoms reaching an 1.22 Å RMSD [29,30].

The compound with the highest binding affinity was found to be **5b** (ΔG = −9.3 kcal/mol, when using AutoDock Vina and ΔG = −10.19 kcal/mol when using AutoDock), while compounds **5a** and **5c** had significantly lower binding affinities. All compounds had lower binding affinities to VEGFR-2 when compared to sorafenib. Even though the two pieces of software have different working principles [31,32,33,34], upon analyzing the overall results, a good correlation was found (R^2^ = 0.92).

**Figure 4:**
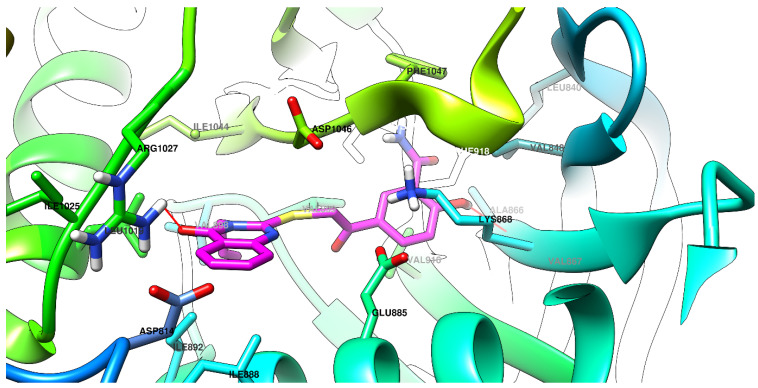
The top binding conformation of **5a** at the ATP-binding site of VEGFR-2 (carbon atoms in magenta, oxygen atoms in red, hydrogen atoms in white, nitrogen atoms in blue, sulfur atom in yellow).

**Figure 5:**
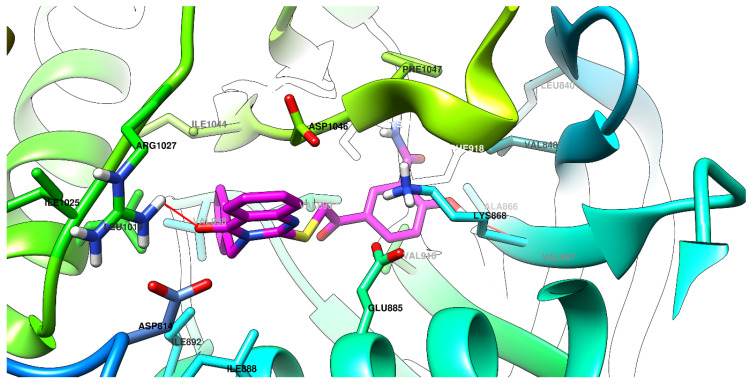
The top binding conformation of **5b** at the ATP-binding site of VEGFR-2 (carbon atoms in magenta, oxygen atoms in red, hydrogen atoms in white, nitrogen atoms in blue, sulfur atom in yellow).

**Figure 6:**
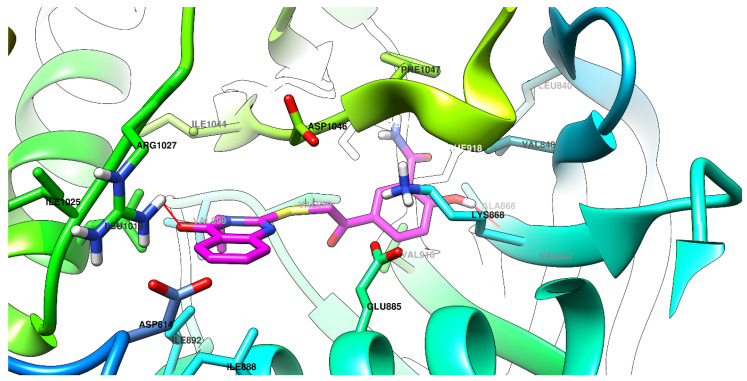
The top binding conformation of **5c** at the ATP-binding site of VEGFR-2 (carbon atoms in magenta, oxygen atoms in red, hydrogen atoms in white, nitrogen atoms in blue, sulfur atom in yellow).

**Figure 7:**
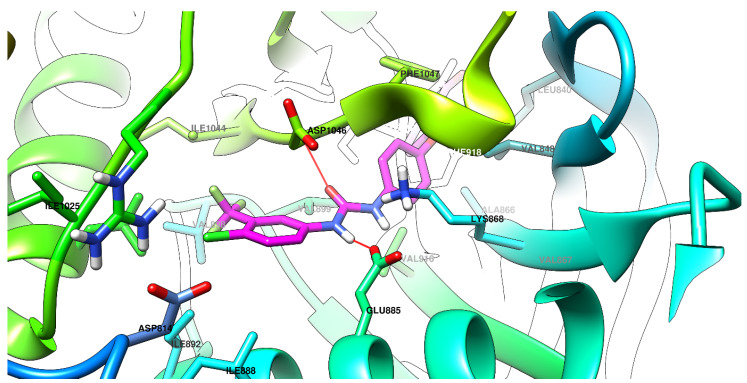
The top binding conformation of **sorafenib** at the ATP-binding site of VEGFR-2 (carbon atoms in magenta, oxygen atoms in red, hydrogen atoms in white, nitrogen atoms in blue, sulfur atom in yellow).

Graphical depictions of the interactions between the compounds and VEGFR-2 are presented in Figure 4, Figure 5, Figure 6 and Figure 7. An identical binding pattern was identified for all of the top predicted binding conformations of compounds **5a**–**c**, where the oxygen atom from quinazolinone was predicted to interact with the positively charged sidechain of Arg1027, while the hydroxyl group from the salicylamide moiety was predicted to be involved in a hydrogen bond as donor (HBD) with the Val867-Ala866 peptide bridge. The variable hydrophobic substituent found in position 3 of the quinazolinone core of compounds **5a**–**c** fitted well in the hydrophobic pocket surrounded by the sidechains of Ile892, Val898, Val899, Leu1019, and Ile1044.

**Figure 8:**
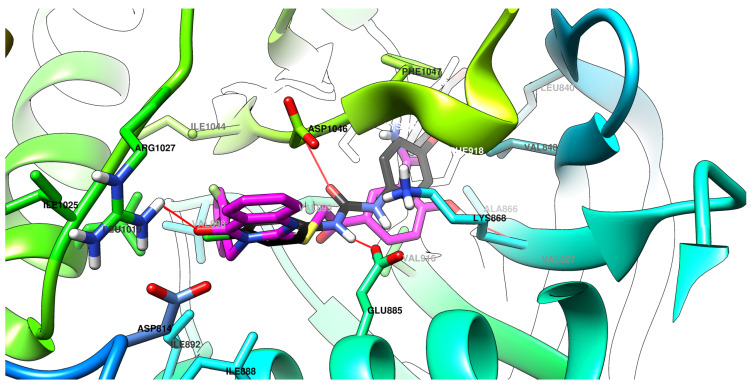
The top superposing binding conformation of compound **5b** and **sorafenib** at the ATP-binding site of VEGFR-2 (carbon atoms in magenta for compound **5b** and in black for **sorafenib**, oxygen atoms in red, hydrogen atoms in white, nitrogen atoms in blue, sulfur atom in yellow).

Graphical depictions of the interactions between compound **5b** superimposed with sorafenib and VEGFR-2 are presented in Figure 8. For the other two compounds, **5a** and **5c**, these interactions are presented in Figures S13 and S14 in the Supplementary Materials. The main highlighted interactions were the same, as shown in Figure 4, Figure 5, Figure 6 and Figure 7. Sorafenib is known as a target tyrosine kinase inhibitor, and can negatively influence antitumoral activity because of its severe side effects and the rapid development of tumor resistance [35]. A different method of binding at the VEGFR-2 active site can also influence the capacity of compounds **5a**–**c** to mitigate side effects and tumor resistance.

In line with the SAR hypothesis, the most important functional groups remained the oxygen atom from quinazolinone, the hydroxyl group from the salicylamide moiety, and the bulkier hydrophobic substituent found on position 3 of the quinazolinone heterocycle.

### 2.3. Molecular Dynamics

Performing molecular dynamics simulations after molecular docking is crucial for validating the docking results and gaining a more realistic, dynamic understanding of the ligand–protein interaction [36].

**Figure 9:**
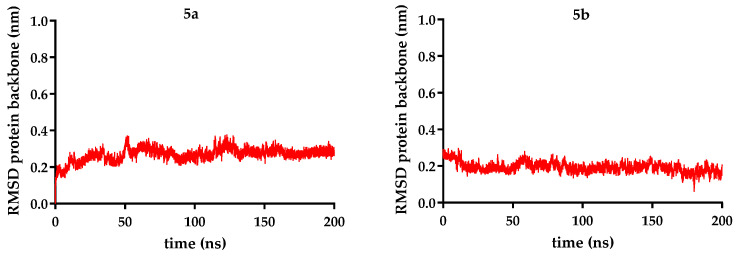

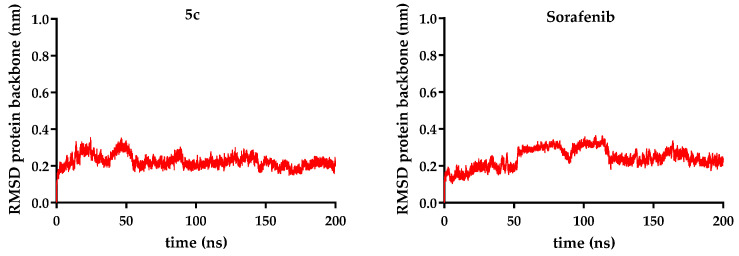
RMSD (root-mean-square deviation) protein backbone (nm) is represented for each of compounds **5a**–**c** over 200 ns, compared to **sorafenib**.

**Figure 10:**
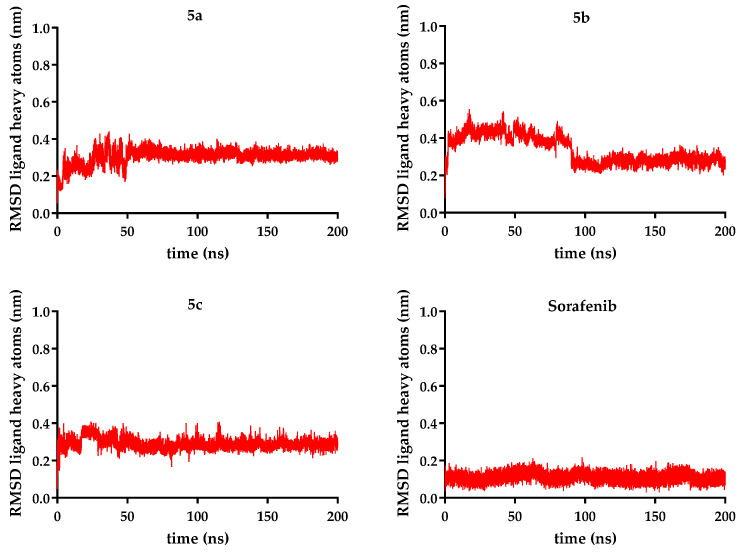
RMSD (root-mean-square deviation) for ligand heavy atoms (nm) is represented for each of compounds **5a**–**c** over 200 ns, compared to **sorafenib**.

**Figure 11:**
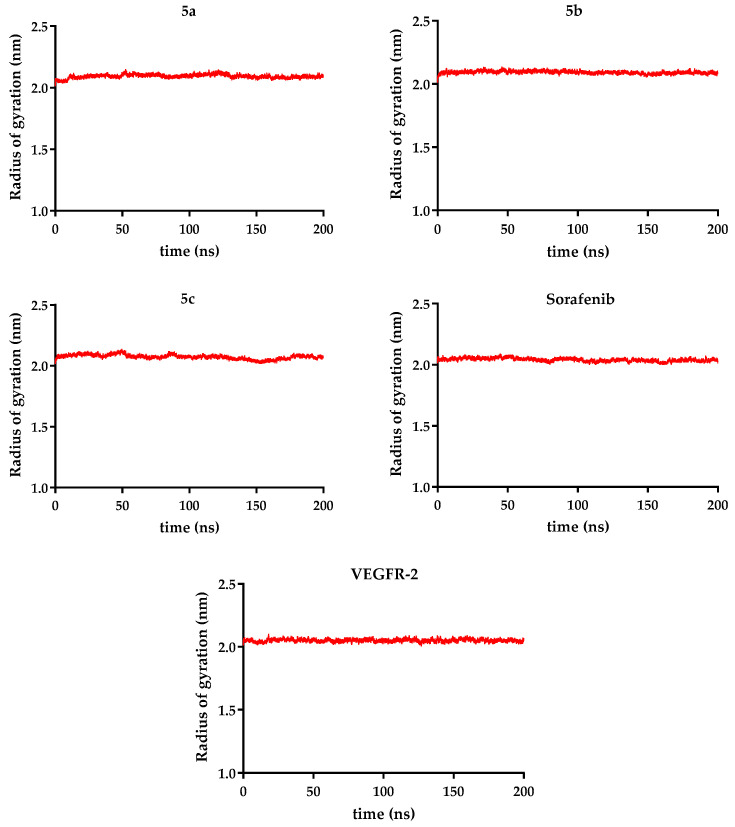
Counting of hydrogen bonds (1 ns moving average) between compounds **5a**–**c** and VEGFR-2 over 200 ns, compared to **sorafenib**.

**Figure 12:**
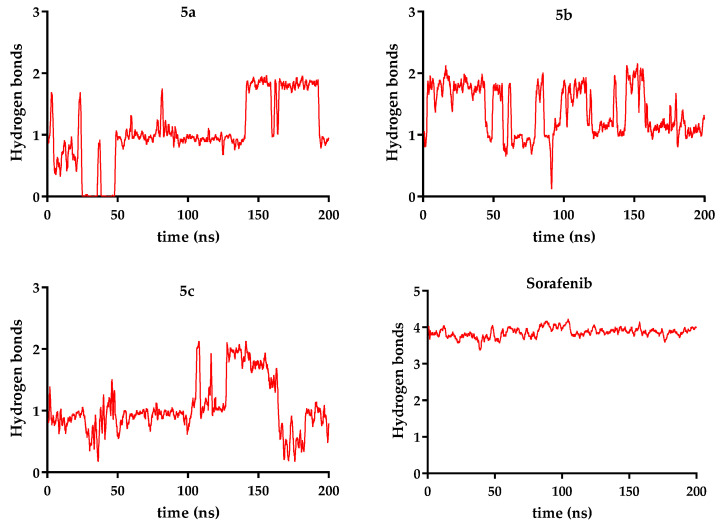
Hydrogen bonds are presented for compounds **5a**–**c** over 200 ns, compared to **sorafenib**.

After performing molecular dynamics studies on the constructed complexes of compounds **5a**–**c** and sorafenib, their trajectories were studied, with the generated summary of data being presented in Table 2. The plotted data resulting from the analysis are also presented (Figure 9, Figure 10, Figure 11 and Figure 12).

The molecular dynamics simulations revealed that all VEGFR-2 complexes maintained structural stability during the simulation period. The backbone RMSD values for the receptor ranged from 0.19 to 0.26 nm, indicating minimal deviation from the starting conformation, with an RMSD of 0.19 nm in the case of the complex with compound **5b**, the lowest RMSD in the current series. Similarly, the receptor’s radius of gyration (2.04–2.09 nm) remained constant across all systems, suggesting that ligand binding did not induce significant protein compaction or expansion.

Root-mean-square fluctuations of α-carbons (0.11–0.12 nm) and amino acid sidechains (0.16–0.17 nm) were comparable among all complexes, implying that local flexibility was not substantially altered upon ligand binding.

Ligand stability within the binding site varied more noticeably. Sorafenib exhibited the lowest ligand RMSD (0.11 nm), confirming its highly stable positioning. In contrast, the novel analogs showed higher fluctuations: compound **5c** (R = ethyl − 0.28 nm) was the most stable of them, while compound **5b** (R = benzyl − 0.33 nm) was the most mobile, when the data were analyzed as an overall average. Taking a deeper look into the trajectory of the ligand, it can be seen that the average did not correctly describe its stability. After significant movement in the first part of the simulation (the first 90 ns), a period when compound **5b** exhibited significant conformational changes, a plateau state was reached in a stable conformation, with a ligand RMSD of 0.27 nm (Figure 9, Figure 10 and Figure 11).

Hydrogen bonding patterns further highlight sorafenib’s superior binding performance, forming an average of 3.86 hydrogen bonds/ns, exceeding the values observed for compound **5a** (R = allyl − 1.05), compound **5b** (R = benzyl − 1.37), and compound **5c** (R = ethyl − 1.04) (Figure 12). Additionally, a supplementary table (Table S1) presenting the number of hydrogen bonds between VEGFR-2 and compounds **5a**–**c**, reported every 10 ns for each compound compared to sorafenib, is included in the Supplementary Materials.

These findings suggest that although compounds **5a**–**c** can stably associate with VEGFR-2 without destabilizing the receptor, they establish fewer hydrogen bonds and display greater mobility compared to sorafenib. Among the tested analogs, compound **5b** demonstrates the most promising interaction profile based on its combination of low receptor RMSD and moderate hydrogen bonding. Further structural optimization is needed to enhance binding stability and affinity.

### 2.4. Molecular Mechanics–Poisson–Boltzmann Surface Area (MM-PBSA)

The Molecular Mechanics–Poisson–Boltzmann Surface Area (MM-PBSA) method is a powerful and widely used computational technique for calculating the binding free energy of molecular complexes, such as a protein–ligand pair. It is often used to refine and validate results from initial molecular docking experiments by providing a more accurate and comprehensive energetic estimate of the binding interaction [37].

To gain deeper insight into the interactions between the **5a**–**c** series and the VEGFR-2 kinase domain (DFG-out conformation), an MM-PBSA analysis was performed on the final 200 ns of the molecular dynamics simulations, and the resulting predicted values are summarized in Table 3.

Among the new derivatives, compound **5b** displayed the most favorable ΔG (−35.33 kcal/mol), primarily due to its strong van der Waals component. This was the only compound with an aromatic substituent represented by a phenyl ring linked through a methylene group at position 3 of the quinazolinone core (−53.01 kcal/mol), providing flexibility to the molecule to achieve better interaction with the active site of VEGFR-2. However, its electrostatic contribution could be attributed to the bulkier substituent from position 3 of the quinazolinone heterocycle, where the π electrons were delocalized, leading to a significantly weaker value (−7.32 kcal/mol) than sorafenib’s (−39.67 kcal/mol). Compounds **5a** and **5c** showed comparable binding energies (−32.29 and −32.64 kcal/mol, respectively), with van der Waals terms (−45.45 kcal/mol each) and moderate electrostatic contributions (−17.35 and −14.81 kcal/mol), due to the smaller substituents in position 3 of the quinazolinone nucleus (allyl and ethyl), where effects of π electron delocalization from an aromatic ring did not occur.

The reference inhibitor sorafenib exhibited a markedly stronger binding affinity (ΔG = −69.39 kcal/mol) compared with the novel compounds **5a**–**c** (−32.29 to −35.33 kcal/mol). The decomposition of energetic contributions revealed that sorafenib benefited from both more favorable van der Waals interactions (−59.19 kcal/mol) and substantially enhanced electrostatic interactions (−39.67 kcal/mol) relative to **5a**–**c**.

The solvation energies for compounds **5a**–**c** (25.00–30.51 kcal/mol) were similar to that of sorafenib (29.47 kcal/mol), suggesting that solvent effects did not significantly differentiate their binding profiles.

**Figure 13:**
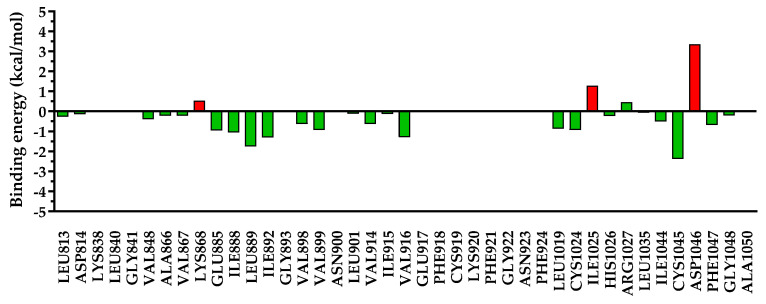
The energy decomposition for amino acids found within 5 Å of **5a**. In green are depicted the favorable interactions, while in red are depicted the unfavorable interactions.

**Figure 14:**
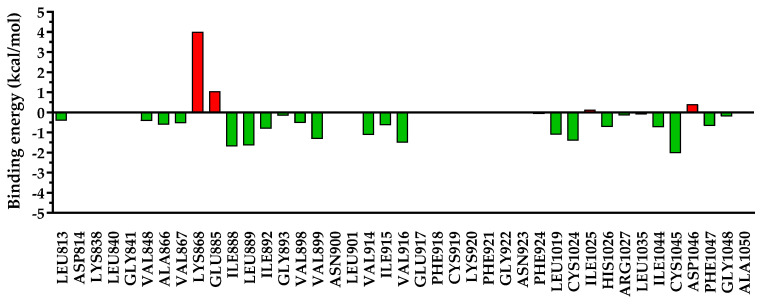
The energy decomposition for amino acids found within 5 Å of **5b**. In green are depicted the favorable interactions, while in red are depicted the unfavorable interactions.

**Figure 15:**
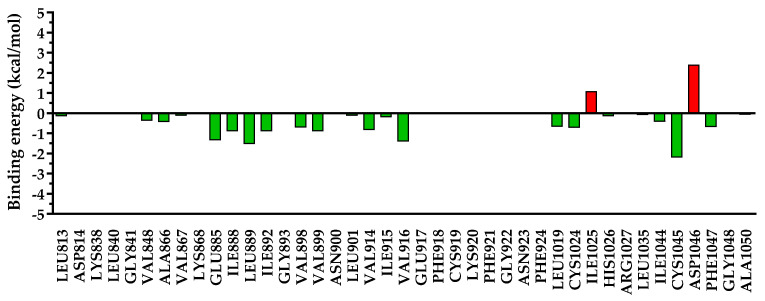
The energy decomposition for amino acids found within 5 Å of **5c**. In green are depicted the favorable interactions, while in red are depicted the unfavorable interactions.

**Figure 16:**
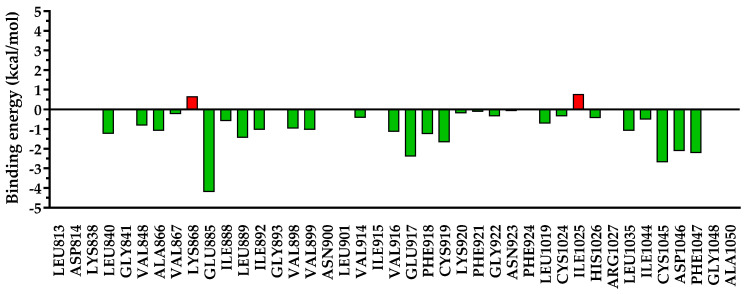
The energy decomposition for amino acids found within 5 Å of **sorafenib**. In green are depicted the favorable interactions, while in red are depicted the unfavorable interactions.

To further elucidate the specific interactions involving amino acids located within 5 Å of the ligands in the complexes, an energy decomposition analysis was carried out, and the corresponding graphical representations are shown in Figure 13, Figure 14, Figure 15 and Figure 16.

Due to the different substitutions of compounds **5a**–**c**, a clear difference could be observed after the decomposition of the interaction energy with the nearby amino acids, especially for undesired repulsive interactions. In the case of compounds **5a** and **5c**, which had a smaller residue in position 3 of the quinazolinone core, a significant repulsion was found with Ile1025 and Asp1046, and a low-to-negligible repulsion with Lys868. On the other hand, in the case of compound **5b** with a bulkier substituent in position 3 of the quinazolinone, the repulsion with Ile1025 and Asp1046 was almost negligible, but it heavily increased the repulsion with Lys868 and moderately increased that with Glu885. In the case of sorafenib, used as a reference agent, only two minor repulsions were identified—with Lys868 (+0.67 kcal/mol) and Ile1025 (+0.78 kcal/mol).

### 2.5. Density Functional Theory (DFT)

In drug design, the electronic properties of newly synthesized compounds play a critical role in determining their chemical reactivity, stability, and potential interactions with biological targets. Among these properties, the energies of the highest occupied molecular orbital (HOMO) and lowest unoccupied molecular orbital (LUMO) are of particular importance. Evaluating the HOMO and LUMO energies of candidate molecules offers valuable predictive information for their pharmacological potential, guiding SAR studies and optimizing lead compounds for therapeutic use. Additionally, the choice of solvent can significantly influence HOMO and LUMO energies by altering the electronic environment through solvation effects, polarization, and stabilization of specific molecular orbitals [38,39].

The HOMO and LUMO energies of compounds **5a**–**c** were evaluated under four dielectric environments—a vacuum, a nonpolar solvent (ε = 7.43), a polar solvent (ε = 37.22), and water—to assess the influence of solvation on their electronic structures (Table 4).

For all three compounds, the HOMO energies showed consistent and modest stabilization as solvent polarity increased. For compound **5a**, the HOMO shifted from –6.18 eV in the vacuum to −6.28 eV in water, and compounds **5b** and **5c** displayed similar trends (−6.19 to −6.26 eV and −6.15 to −6.27 eV, respectively). These shifts, although small (≤0.12 eV), indicated dielectric screening by the solvent, which stabilized the electron-rich frontier orbital.

The LUMO energies also exhibited minor solvent-dependent changes. For compound **5a**, the LUMO increased slightly from −1.82 eV (vacuum) to −1.75 eV (water), suggesting weak destabilization with rising polarity. Compounds **5b** and **5c** showed analogous patterns, with LUMO values varying within ~0.09 eV across all conditions. Importantly, the absence of abrupt changes or anomalies implied that solvation effects were uniform and predictable for this compound series.

The HOMO–LUMO gaps remained narrow and stable, ranging from ~4.43 to 4.53 eV, regardless of the solvent environment. This invariance suggests that the electronic reactivity, redox potential, and overall stability of these molecules were largely unaffected by dielectric constant—an advantageous property for potential pharmaceutical applications, where compounds encounter diverse environments (e.g., hydrophobic binding pockets versus aqueous environment). The slight stabilization of the HOMO levels likely arose from nonspecific dielectric polarization rather than strong solute–solvent interactions such as hydrogen bonding.

In all **5a**–**c** compounds, HOMO was found over the quinazolinone region of the compounds, while LUMO was found over the salicylamide region of the compounds (Figures S15–S20 from Supplementary Materials). The distribution of the electrons across the molecules was similar across compounds **5a**–**c**, with electron-rich regions near oxygen atoms, with these electrons able to participate in hydrogen bonding as acceptors or nucleophilic interactions, while electron-deficient regions appeared around amide hydrogen atoms, indicating a potential site for an electrophilic attack or hydrogen-bond donation. The predominance of green across the aromatic rings suggested a largely nonpolar, hydrophobic character in these areas, which might favor π–π stacking, π–cation interactions, or van der Waals interactions, important types of noncovalent interactions with biological targets [40,41,42] (Figures S21–S23 from Supplementary Materials). Overall, the molecular electrostatic potential (MEP) map suggests a molecule with localized polar sites suitable for specific binding interactions, combined with extensive hydrophobic surfaces that could stabilize binding within nonpolar pockets of a receptor or enzyme. This distribution supported the balance of polarity and lipophilicity inferred from the LogP and TPSA values, reinforcing the compound’s potential as a drug-like candidate.

The obtained results demonstrated that compounds **5a**–**c** maintained robust electronic characteristics across media of varying polarity. This electronic resilience supported their candidacy as drug-like molecules, as their electronic properties—and thus, their reactivity and potential biological interactions—were unlikely to fluctuate significantly under physiological conditions.

The conformations that represent the HOMO and the LUMO parameters for each compound, as well as an electrostatic potential map of them, are presented in the Supplementary Materials (Figures S13–S21).

Some important molecular properties were computed and are presented in Table 5. The molecular surface area and volume followed a similar trend, with compound **5b** exhibiting the largest values (438.03 Å^2^ and 425.95 Å^3^), implying a bulkier structure that could influence steric fit in biological targets.

Notably, compound **5c** showed the highest dipole moment (2.60 D), indicating increased polarity that might enhance specific interactions with polar residues at a binding site.

Together with the frontier orbital data, these parameters suggested that compound **5b** could prefer hydrophobic regions, compound **5a** had an intermediate profile, while compound **5c** might benefit from stronger electrostatic interactions due to its higher dipole moment.

These combined electronic and physicochemical characteristics were essential for predicting pharmacokinetic behavior and target engagement, reinforcing the potential of compounds **5a**–**c** as drug-like candidates with distinct yet complementary properties.

### 2.6. Cytotoxicity Assay

**Figure 17:**
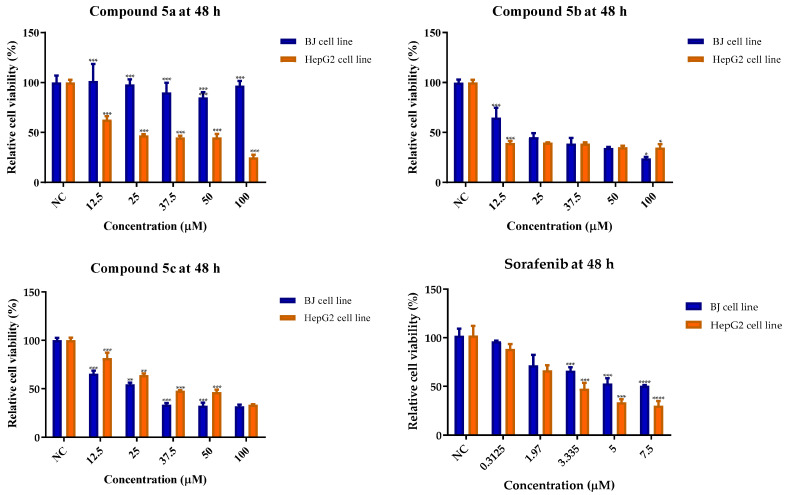
The cytotoxic effects of **5a**–**c** and **sorafenib** on BJ and against HepG2 cells, after 48 h of cell exposure. The results are presented as relative means ± standard deviations (SD) of three biological replicates (six technical replicates for each biological replicate). Viability data are expressed as relative values, where the negative control (NC) was set at 100%. * *p* < 0.05, ** *p* < 0.01, and *** *p* < 0.001, **** *p* < 0.0001, compared to the negative control (NC) group.

The cytotoxic effects of **5a**–**c** were evaluated on normal fibroblast cells (BJ) and against one cancer cell line, human hepatocellular carcinoma (HepG2), at 48 h after cell exposure. Sorafenib was used as a reference agent. The IC_50_ values (μM) and the selectivity index (SI) are presented in Table 6. The relative cell viability (%) is depicted in Figure 17.

Concerning the BJ cell line, no IC_50_ value could be calculated in the range of tested concentrations for the **5a** compound. It could be considered less cytotoxic or non-cytotoxic on this cell line. The second compound, **5b**, had the lowest IC_50_ value, exhibiting moderate cytotoxicity and affecting the BJ cells less than sorafenib. The third compound, **5c**, was less harmful to healthy cells than **5b** and the positive standard, also indicating a moderate cytotoxicity effect.

As in the previous case, the IC_50_ values corresponding to **5a** could not be determined based on the results obtained on the HepG2 cell line. This could indicate that it might not possess antitumor activity against hepatocellular carcinoma. Compound **5b** exhibited the highest potency against HepG2 cells, but did not have better IC_50_ values than sorafenib. Compound **5c** showed less cytotoxic potential than **5b** and sorafenib. Both **5b** and **5c** exhibited antitumor activity against the HepG2 cell line, demonstrating higher cytotoxicity compared to **5a**, as indicated by the IC_50_ values.

Sorafenib, the reference agent, was more cytotoxic on both BJ and HepG2 cell lines than the synthesized compounds. Its antitumoral activity on HepG2 cells is well-documented in the literature, and it is used as a first-line treatment for hepatocellular carcinoma [43].

The obtained SI values for compounds **5b** and **5c** were superior to that for sorafenib. Neither of them was greater than 2, which is considered suitable in the literature [30].

Regarding the relative cell viability values after 48 h of cell exposure, compound **5a** had lower cytotoxicity on the BJ cell line at all tested concentrations, and had a better profile than sorafenib. Compound **5b** decreased the cell viability by more than 50%, especially at concentrations over 25 µM, but reduced the viability values by more than 35% at concentrations higher than 50 and 100 µM. The obtained results for **5c** were significantly unfavorable over 37.5 µM. Sorafenib did not reduce relative cell viability by more than 50% at any of the tested concentrations.

With regard to cellular viability on the HepG2 line, compound **5a** affected it by at least 50% starting from a concentration of 25 µM, but was not as potent as sorafenib at 5 µM. Compound **5b** influenced viability by more than 50% over a concentration of 12.5 µM; while it was not superior to sorafenib, it was the most active compound against this cell line. Compound **5c** led to an important reduction in cell viability over a concentration of 37.5 µM, being less active on HepG2 cells. Sorafenib showed cell viability levels below 50% at concentrations exceeding 5 µM.

In the context of evaluating antitumor activity on the HepG2 cell line, structure–activity relationship (SAR) analysis revealed that the introduction of an aliphatic substituent (**5a**, R = allyl, or **5c**, R = ethyl) in the third position of the quinazolin-4-one scaffold had an unfavorable impact on cytotoxicity. In contrast, replacement with a bulkier substituent (**5b**, R = benzyl) in the same position led to increased cytotoxicity. All the tested compounds had a thioether and a ketone group in the second position of the quinazolin-4-one heterocycle, which was included in a linker, and continued with a salicylamide moiety [5,6,16].

The unsaturated aliphatic substituent in position 3 (compound **5a**, R = -H_2_C-CH=CH_2_, a derivative from garlic essential oil known to have antitumor properties [44], and more reactive than ethyl due to the alkene`s presence) exhibited no cytotoxic effects on BJ cells, and did not have the highest potency against the HepG2 cell line. Cytotoxicity was notably influenced by the presence of the benzyl residue in position 3 (compound **5b**, R = -H_2_C-C_6_H_5_, an aromatic moiety with higher flexibility than phenyl), for both cell lines. A saturated aliphatic fragment in position 3 (compound **5c**, R = -H_2_C-CH_3_) showed moderate cytotoxic effects on BJ cells compared to the benzyl residue, and higher cytotoxic effects than the allyl fragment. Against HepG2 cells, the effect of the ethyl residue was superior to that of allyl, but inferior to that of benzyl residue.

### 2.7. ADME Studies

#### 2.7.1. In Silico

In silico models assess a drug’s “drug-likeness” and pharmacokinetic profile, providing insights into its potential bioavailability, metabolic stability, and ability to reach its target [45].

The computed in silico physicochemical descriptors for compounds **5a**–**c** are shown in Table 7. Each compound had a molecular weight between 383.09 and 445.11 g/mol. All three compounds had six or seven rotatable bonds, seven hydrogen bond acceptors, and three hydrogen bond donors. The value of the topological polar surface area (TPSA) was 115.28 Å in all three cases. Additionally, the logP value for each compound was between 2.419 and 3.14, and the logS value was between −4.313 and −4.036. None of the compounds had violations of Lipinski’s rule of five (MW ≤ 500, logP ≤ 5, No. HA ≤ 10, and No. HD ≤ 5) [2,19,27,28].

In silico pharmacokinetic (absorption, distribution, metabolism, and elimination) descriptors are presented in Table 8. The Caco-2 permeability log values for all compounds ranged from −5.467 to −5.380. None of the compounds were predicted to be P-gp substrates or to cross the blood–brain barrier (BBB), but a high plasma protein-binding (PPB) potential was predicted, between 97.9 and 98.7%. The computed metabolization descriptors indicated that all compounds were highly predicted to be CYP2C19 inhibitors. A similar finding was observed for CYP2C9 inhibition, except for compound **5a**, which exhibited a lower probability compared to compounds **5b** and **5c**. Moreover, compounds **5a** and **5c** were found to have a moderate probability of being CYP3A4 inhibitors. No compounds were found to be CYP2D6 inhibitors. Promising human liver microsomal (HLM) stability was predicted only for compound **5c**. The plasma clearance of all three compounds ranged from 4.109 to 4.889 mL/min/kg, while their half-lives was between 0.474 and 0.529 h.

All compounds were predicted to have good druggability properties since they had no violations of Lipinski’s rule of five. The grafted substituents (allyl for **5a**, benzyl for **5b**, and ethyl for **5c**) did not influence the No. HA or No. HD descriptors. The topological polar surface area (TPSA) values were similar for all three molecules (115.28 Å^2^), suggesting comparable hydrogen-bonding capacities and membrane permeability. However, ethyl substitution decreased the No. RB, logP, and MW values, while benzyl substitution increased them. Despite its lower lipophilicity, compound **5c** could be involved in strengthening electrostatic interactions, as our analysis indicated that it has a higher dipole moment.

The pharmacokinetic profile of the proposed compounds was characterized using specific descriptors for all four phases of pharmacokinetics. Concerning absorption, compounds **5a**–**c** were predicted to be poorly absorbed from the gastrointestinal tract, since they were predicted to have low Caco-2 permeability (log value lower than −5.150), and they were not predicted to be P-gp substrates [46]. Additionally, the predicted low absorption is also supported by the values of logS, which indicate that these compounds are only slightly soluble or insoluble in water [47].

Compounds **5a**–**c** were predicted to alter the metabolism of other drugs by inhibiting the most prevalent CYP450 isoenzymes in metabolic processes, specifically CYP3A4, 2C19, and 2C9. A noticeable distinction between the compounds was observed in the case of the HLM stability descriptor. Compound **5c** was the only one predicted to have higher metabolic stability, surpassing the threshold of 30 min, when metabolized by HLM.

Based on the predicted plasma clearance values, all three compounds appeared to have low clearance (<5 mL/min/kg). The predicted half-life values suggested that all three compounds may be drugs with intermediate ultra-short half-lives (under 1 h).

#### 2.7.2. In Vitro Lipophilicity

Lipophilicity is an essential physicochemical descriptor, correlated with both pharmacodynamic and pharmacokinetic properties. It significantly influences membrane permeability, binding to plasma proteins, and interaction with receptors at the active site of action. Therefore, it is considered a key parameter in predicting the biological activity of pharmaceutical compounds. From a physicochemical perspective, lipophilicity is expressed by the logarithmic distribution coefficient between n-octanol and water (logP) [48].

The experimental determination of lipophilicity was carried out using two types of chromatography: reverse-phase thin-layer chromatography (RP-TLC) and reverse-phase high-performance liquid chromatography (RP-HPLC). The values of the retention parameters R_f_ and R_M_ at five different concentrations of isopropanol–water (*v*:*v* %) for compounds **5a**–**c** are presented in Table 9.

The results obtained for the retention parameters (mR_M_ and R_M0_) and for the regression line parameters (R_M_ = f(c)), achieved through RP-TLC, along with the values for the capacity factor log k, acquired via RP-HPLC, are presented in Table 10.

**Figure 18:**
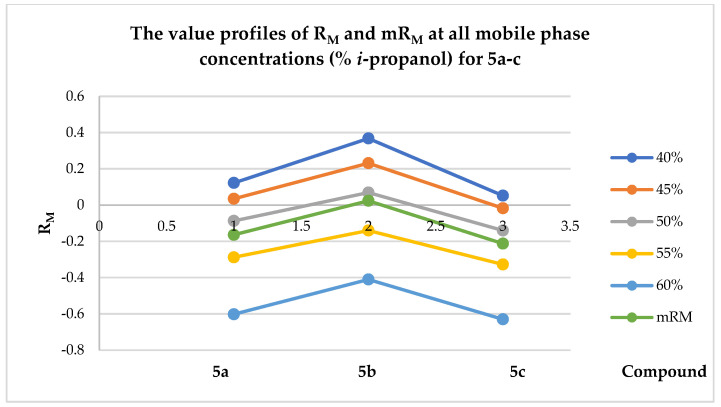
The graphical interpretation of the values of R_M_ and mR_M_ at all mobile-phase concentrations (% i-propanol) for compounds **5a**–**c.**

Starting from the hypothesis that a compound with higher lipophilicity has a greater affinity for the non-polar stationary phase, its profile is characterized by the exhibition of lower R_f_ values and, consequently, higher R_M_ values. The organic modifier used to change the polar mobile phase was isopropanol. It was employed to give a hydrophobic nature to the studied compounds by accelerating the elution and reducing their retention at the stationary phase. Suitable linear dependence (R^2^ > 0.93) was achieved by analyzing the graphical representation of R_M_ values, used as a function of the isopropanol concentrations in the mobile phase (Figure 18). Therefore, based on the line equation corresponding to each compound, the lipophilicity parameter R_M0_ and the slope b were determined (Table 9).

As the values of the *i*-propanol fraction in the mobile-phase composition increased, a decrease in the R_M_ values was observed. Moreover, a similar retention mechanism was highlighted for the quinazolin-4(3*H*)-one derivatives, as evidenced by a good correlation (R^2^ > 0.93) between the R_M0_ values and the slope b. From Figure 18**,** it is clear that compound **5b** was the most lipophilic, while compound **5c** was the least lipophilic. These results were correlated with those obtained by in silico evaluation (Table 7), thus exhibiting the same profile of lipophilicity for the tested compounds.

**Figure 19:**
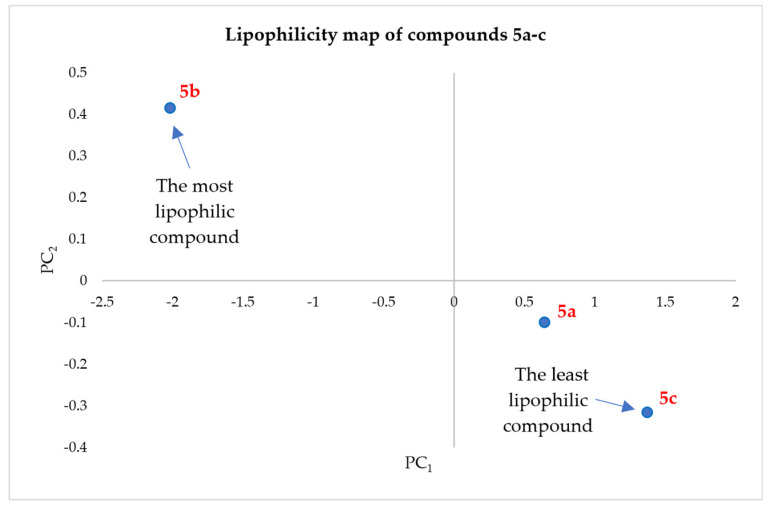
Lipophilicity map of compounds **5a**–**c**, according to PC_1_ and PC_2_ values.

The principal component analysis (PCA) method was applied to the initial dataset, including the R_M_ values for all five applied mobile phases, in order to reduce the large number of variables to those that retain the initial information. Lipophilicity maps were obtained to graphically represent the information obtained from the two principal components (Figure 19). Concerning final compounds **5a**–**c**, the first principal component PC_1_ accounted for 91.34% of the data variability, while the second principal component PC_2_ represented an additional 0.16% [49].

The values of the two parameters PC**_1_** and PC**_2_** for compounds **5a**–**c** are presented in Table 11. A lower value of the PC**_1_** parameter suggests a more lipophilic compound. Through the obtained results, a good linear correlation was established between R_M0_ and PC**_1_** for the quinazolin-4(3*H*)-one derivatives **5a**–**c** (R^2^ = 0.979).

In the case of compounds **5a**–**c**, the most lipophilic compound was **5b**, in which the quinazolin-4(3*H*)-one heterocycle was substituted with a benzyl radical in position 3, and the last lipophilic compound was **5c**, in which the quinazolin-4(3*H*)-one heterocycle was substituted with an ethyl radical in position 3. Both compounds were substituted in position 2 of the heterocycle with the *N*-thioacetyl-2-hydroxybenzamide residue. Therefore, the lipophilicity was influenced by the substituent from position 3, an aromatic residue such as a benzyl, which is more hydrophobic than an unsaturated aliphatic allyl fragment or a saturated aliphatic ethyl moiety.

The log k parameter was calculated for compounds **5a**–**c** using the retention time values determined by the RP-HPLC method, and the results are presented in Table 9 [49].

The highest retention time values were recorded for compound **5b**, which contains a benzyl moiety in position 3 of the quinazolin-4(3*H*)-one heterocycle, and the lowest values were recorded for compound **5c**, which holds an ethyl residue in the same position.

The results obtained by RP-HPLC were consistent with those obtained by the RP-TLC method. For final compounds **5a**–**c**, values of lipophilicity followed the obtained values for the LogP parameter on the ADMETlab 3.0 online platform [50]. Both applied calculation methods showed that the lowest values of the lipophilicity parameters were obtained for compound **5c,** and the highest values for compound **5b**.

The correlation between the experimental and theoretical values of the lipophilicity parameters was analyzed for compounds **5a**–**c** (Table 12).

The calculated correlation coefficients ranged from −0.97 to 0.99. The correlation between the obtained in silico logP values and the experimentally determined lipophilicity parameters was in the range of −0.99 to 0.99. A high correlation was found between the experimentally examined parameters R_M0_ and PC_1_ (0.97). Additionally, the values of the correlation coefficients between log k, obtained by RP-HPLC determinations, and mR_M_, R_M0_, and PC_1_, determined by the RP-TLC method, are 0.99, −0.98, and −0.99, respectively.

## 3. Materials and Methods

### 3.1. Chemical Synthesis

For the chemical synthesis, purification, structural analysis, and in vitro cytotoxicity evaluation, reagents were purchased from local suppliers and Merck KGaA (Darmstadt, Germany), and used according to the instructions.

Analytical thin-layer chromatography (TLC) was performed on Silica Gel 60 F_254_ sheets to monitor the reaction progress and to verify the purity of the tested compounds. The elution system employed was a mixture of ethyl acetate and n-hexane in a 3:1 ratio. The visualization was carried out with UV light at 254 nm.

Based on the glass capillarity method, melting points were measured using a melting point device MPM-H1 (Schorpp Gerätetechnik, Überlingen, Germany).

The structures of compounds **5a**–**c** were confirmed by recording and examining the IR, MS, ^1^H-NMR, and ^13^C-NMR spectra. The IR spectra were recorded with an FT/IR 6100 spectrometer (Jasco, Cremella, Italy) using KBr pellets. To record the MS spectra, an Agilent Ion Trap SL mass spectrometer instrument (Agilent Technologies, Santa Clara, CA, USA) was used in positive ionization mode for final compounds **5a**–**c**. ^1^H-NMR and ^13^C-NMR spectra, operating at 500 MHz and 125 MHz, were recorded using an Avance NMR spectrometer (Bruker, Karlsruhe, Germany) in dimethylsulfoxide-*d*_6_ (DMSO-*d*_6_), and calibration of the spectrometer was carried out using tetramethylsilane (TMS). The chemical shift (δ) values are described in parts per million (ppm). The following abbreviations for peak patterns were used to identify the multiplicity of the signals in the ^1^H-NMR spectra: s—singlet, d—doublet, dd—doublet, td—triplet of doublets, and m—multiplet. For the signals given by the hydrogen or carbon atoms, to describe the location of the atom in a specific region of the molecule, the following abbreviations are used: Q—quinazolin-4(3*H*)-one and Ar—hydroxybenzamide ring.

*General procedure for the synthesis of intermediate compounds ***3a**–**c**

Compounds **3a**–**c** were synthesized using a protocol previously reported by our group and presented in Scheme 1 [27,28].

*General procedure for the synthesis of final compounds ***5a**–**c**

In a glass flask, 3 mmol of compounds **2a**–**c** and 2 mmol of potassium carbonate were added to 5 mL of dimethylformamide (DMF). The mixture was stirred at room temperature (RT) for 2 h. A total of 3 mmol of 5-(2-bromoacetyl)-2-hydroxybenzamide was added. The mixture was stirred at room temperature for 4–6 h until TLC indicated total consumption of intermediate compounds **3a**–**c**. The resulting precipitate was obtained by pouring water on it and neutralizing it with a 10% hydrochloric acid solution. Final compounds **5a**–**c** were filtered under vacuum conditions, dried, and crystallized from dioxane.

The structural assignments of **5a**–**c** were determined using their IR, MS, ^1^H-NMR, and ^13^C-NMR spectral data.

*5-(2-((3-allyl-4-oxo-3,4-dihydroquinazolin-2-yl)thio)acetyl)-2-hydroxybenzamide (**5a**):* white solid; mp = 227 °C; yield = 78%; FT IR (KBr) ν_max_cm^−1^: 1688.37, 1678.73, 1659.45 (str C=O), 1618.95 (N-H), 1550.49 (C=N), 1319.07 (Ar-OH), 1209.63 (C-N); MS: *m/z* = 396.30 [M+H^+^]; ^1^H-NMR (DMSO-*d*_6_, 500 MHz) δ: 8.76 (d, 2H, Ar-CONH_2_, ^2^*J*H-H = 2.00 Hz), 8.14 (dd, 2H, H-2, H-6, Ar, ^1^*J*H-H = 8.50 Hz, ^2^*J*H-H = 2.50 Hz), 8.04 (dd, 1H, H-5, Q, ^1^*J* = 8.00 Hz, ^1^*J*H-H = 1.00 Hz), 7.67 (td, 1H, H-6, Q, ^1^*J*H-H = 7.75 Hz, ^1^*J*H-H = 1.75 Hz), 7.39 (td, 1H, H-7, Q, ^1^*J*H-H = 7.75 Hz, ^1^*J*H-H = 2.50 Hz), 7.06 (d, 1H, H-3, Ar, ^1^*J*H-H = 9.00 Hz), 7.03 (d, 1H, H-8, Q, ^1^*J*H-H = 8.00 Hz), 6.02–5.94 (m, 1H, -CH=), 5.26 (dd, 1H, =CH*_2_*, ^1^*J*H-H = 10.25 Hz, ^1^*J*H-H = 1.25 Hz), 5.17 (dd, 1H, =CH_2_, ^1^*J*H-H = 17.00, ^1^*J*H-H = 1.00 Hz), 4.85 (s, 2H, -CH_2_-), 4.78 (d, 2H, -CH_2_-, ^2^*J*H-H = 5.50 Hz); ^13^C-NMR (DMSO-*d*_6_, 125 MHz) δ: 191.38 (C=O), 171.54 (C=O), 165.44 (Ar-OH), 160.17 (C=O), 156.14 (C=N), 146.51 (Q), 134.69 (Ar), 134.05 (Q), 131.30 (-CH=), 129.84 (Ar), 127.40 (Ar), 126.44 (Q), 125.92 (Q), 125.44 (Q), 118.53 (=CH_2_), 117.93 (Q), 117.51 (Q), 113.96 (Ar), 46.02 (-CH_2_-), 38.51 (-CH_2_-).

*5-(2-((3-benzyl-4-oxo-3,4-dihydroquinazolin-2-yl)thio)acetyl)-2-hydroxybenzamide (**5b**):* white solid; mp = 267 °C; yield = 72%; FT IR (KBr) ν_max_cm^−1^: 1685.48, 1661.37, 1607.86 (str C=O), 1618.95 (N-H), 1547.59 (C=N), 1319.07 (Ar-OH), 1206.26 (C-N); MS: *m/z* = 446.30 [M+H^+^]; ^1^H-NMR (DMSO-*d*_6_, 500 MHz) δ: 8.76 (d, 2H, Ar-CONH_2_, ^2^*J*H-H = 2.00 Hz), 8.13 (dd, 2H, H-2, H-6, Ar, ^1^*J*H-H = 8.75 Hz, ^2^*J*H-H = 1.75 Hz), 8.07 (dd, 1H, H-5, Q, ^1^*J*H-H = 8.00 Hz, ^1^*J*H-H = 1.00 Hz), 7.69 (td, 1H, H-6, Q, ^1^*J*H-H = 7.50 Hz, ^2^*J*H-H = 1.75 Hz), 7.41 (td, 1H, H-7, Q, ^1^*J*H-H = 7.75 Hz, ^2^*J*H-H = 1.00 Hz), 7.38–7.27 (m, 5H, Bz), 7.05 (t, 2H, H-8, Q, ^1^*J*H-H = 8.50 Hz), 5.40 (s, 2H, =CH_2_), 4.85 (s, 2H, -CH_2_-); ^13^C-NMR (DMSO-*d*_6_, 125 MHz) δ: 191.32 (C=O), 171.54 (C=O), 165.44 (Ar-OH), 160.68 (C=O), 156.36 (C=N), 146.52 (Q), 134.58 (Bz), 134.82 (Ar), 134.04 (Q), 129.82 (Ar), 128.54 (Bz), 127.42 (Ar), 127.37 (Bz), 126.78 (Bz), 126.56 (Q), 126.01 (Q), 125.47 (Q), 118.53 (Bz), 117.91 (Q), 113.93 (Ar), 47.13 (-CH_2_-), 38.55 (-CH_2_-).

*5-(2-((3-ethyl-4-oxo-3,4-dihydroquinazolin-2-yl)thio)acetyl)-2-hydroxybenzamide (**5c**):* white solid; mp = 236 °C; yield = 71%; FT IR (KBr) ν_max_cm^−1^: 1672.95, 1654.62, 1577.49 (str C=O), 1614.61 (N-H), 1552.42 (C=N), 1314.73 (Ar-OH), 1202.88 (C-N); MS: *m/z* = 384.20 [M+H^+^]; ^1^H-NMR (DMSO-*d*_6_, 500 MHz) δ: 8.77 (d, 2H, Ar-CONH_2_, ^2^*J*H-H = 2.00 Hz), 8.14 (dd, 2H, H-2, H-6, Ar, ^1^*J*H-H = 8.75 Hz, ^2^*J*H-H = 2.50 Hz), 8.04 (dd, 1H, H-4, Q, ^1^*J*H-H = 8.00 Hz, ^2^*J*H-H = 1.00 Hz), 7.66 (td, 1H, H-5, Q, ^1^*J*H-H = 7.50 Hz, ^2^*J*H-H = 1.50 Hz), 7.39 (td, 1H, H-6, Q, ^1^*J*H-H = 7.75, ^2^*J*H-H = 1.00 Hz), 7.06 (d, 1H, H-7, Ar, ^1^*J*H-H = 8.50 Hz), 7.01 (d, 1H, H-8, Q, ^1^*J*H-H = 8.00 Hz), 4.86 (s, 2H, -CH_2_-), 4.19–4.15 (m, 2H, -CH_2_-), 1.34 (t, 3H, -CH_3_, ^3^*J*H-H = 7.00 Hz); ^13^C-NMR (DMSO-*d*_6_, 125 MHz) δ: 191.38 (C=O), 171.50 (C=O), 165.49 (Ar-OH), 160.14 (C=O), 155.70 (C=N), 146.49 (Q), 134.55 (Ar), 134.02 (Q), 129.85 (Ar), 127.33 (Ar), 126.31 (Q), 125.92 (Q), 125.83 (Q), 125.37 (Q), 118.66 (Ar), 117.95 (Q), 113.98 (Ar), 39.84 (-CH_2_-), 38.29 (-CH_2_-), 13.01 (-CH_3_).

The spectral data were found to be consistent with the proposed structures of synthesized compounds **5a**–**c**.

All the representative signals were detected in the IR spectra. Three strong C=O stretching signals were found between 1577.49 and 1688.37 cm^−1^: one from the quinazolin-4(*3H*)-one heterocycle and two from the 5-thioacetyl-2-hydroxybenzamide residue. The C=N stretching, from the quinazolin-4(*3H*)-one heterocycle, had a specific signal at 1547.59–1552.42 cm^−1^. From the 2-hydroxybenzamide fragment, the N-H stretching had a signal at 1614.61–1618.95 cm^−1^, the Ar-OH stretching had a specific signal at 1314.73–1319.07 cm^−1^, and the C-N stretching a specific signal at 1202.88–1209.63 cm^−1^. All the identified signals proved that the chemical synthesis took place successfully.

In the MS spectra of **5a**–**c**, the identified molecular mass peaks were in accordance with the chemical structures.

In the ^1^H-NMR spectra of **5a**–**c**, all expected proton signals were identified. In the ^13^C-NMR spectra, the expected signals corresponding to the carbon atoms were identified, and all the signals were observed within the anticipated spectral regions.

Graphic depictions of the recorded IR, MS, ^1^H-NMR, and ^13^C-NMR spectra for compounds **5a**–**c** are presented in the Supplementary Materials (Figures S1–S12).

### 3.2. Molecular Docking

The structure of human VEGFR-2 co-crystallized with sorafenib was taken from the Protein Data Bank (PDB code 4ASD) [51,52]. The processing of the targeted protein was reported in our previous paper, and preparation for molecular docking targeting was carried out using AutoDockTools 1.5.6 [30,53,54].

The molecular docking study targeted the binding site of ATP through inclusion of the co-crystallized sorafenib in the configured search space. The search space was centered on x = −21.966, y = −0.473, and z = −11.442, according to our previously reported protocol. The molecular docking study was performed using AutoDock Vina 1.1.2 as the main software, while AutoDock 4.2 was used as secondary software for confirmation of the results [30,32,53].

The files containing the 3D structures of compounds **5a**–**c** and sorafenib as a reference compound were generated using Avogadro 1.2.0 and processed further using AutoDockTools 1.5.6 [53,55]. Visualization of the conformations resulting from the molecular docking was carried out using UCSF Chimera 1.10.2 [56].

### 3.3. Molecular Dynamics

For compounds **5a**–**c**, the docking conformation with the highest binding affinity was used to construct chimeric complexes with VEGFR-2, which were subsequently evaluated through molecular dynamics simulations. Simulations were conducted with GROMACS 2024.5 by employing the CHARMM36 force field and TIP4P water model in an orthorhombic box [57,58,59]. Ligand topologies were generated using CGenFF, and system preparation—comprising solvation, counterion neutralization, and energy minimization—following established protocols [60,61].

Each complex was simulated for 200 ns to assess structural stability and conformational dynamics. Calculations were performed on a Debian Linux 12 system equipped with an AMD Ryzen™ 9 7900 CPU and an NVIDIA RTX 3060 GPU with CUDA 12.8. The trajectory analyses were visualized using VMD 1.9.4, and analysis parameters were computed using the GROMACS built-in functions [62].

### 3.4. Molecular Mechanics–Poisson–Boltzmann Surface Area (MM-PBSA)

The free binding energies for ligands **5a**–**c** and sorafenib with VEGFR-2 were estimated using the Molecular Mechanics–Poisson–Boltzmann Surface Area (MM-PBSA) method, carried out with gmx_MMPBSA, following procedures previously described by our group [30,63]. The evaluation was made in the last 200 ns of each molecular dynamics simulation, equivalent to 20.000 frames. To clarify the energetic contributions of individual amino acids within 5 Å of the ligand, an energy decomposition analysis was conducted. The binding free energy between the ligand and VEGFR-2 was then determined using the following relationship [64,65]:

ΔG_binding = G_complex − (G_protein + G_ligand),(1)

### 3.5. Density Functional Theory (DFT)

The DFT in silico calculations were performed with the B3LYP-D3 functional method and the 6-311G(2d,p) basis set using Spartan 24 1.1.0 (Wavefunction, Inc., Irvine, CA, USA) to investigate the electronic and structural characteristics of compounds **5a**–**c**. The calculations were performed to identify the regions of the molecules that are important for their interaction with VEGFR-2 according to their electron density, frontier molecular orbitals, and dipole moment.

### 3.6. Cytotoxicity Assay

Compounds **5a**–**c** were dissolved in dimethylsulfoxide (DMSO) at an initial concentration of 10 mM, which was diluted 10 times with the characteristic medium and placed on plates at final concentrations ranging from 12.5 to 150 μM. The cells were seeded on plates of 96 wells at a density of 1 × 10^4^ cells per well and incubated for 48 h. The culture medium was removed, and then 100 µL MTT (3-(4,5-dimethylthiazol-2-yl)-2,5-diphenyltetrazolium bromide) was added for 1 h. After that, it was removed, and 150 µL of pure dimethylsulfoxide (DMSO) was added. The samples were read using an ELISA Reader (BioTek, Winooski, VT, USA) at 570 nm. Sorafenib was used as a reference agent at concentrations ranging from 0.03125 to 7.5 μM. All determinations were made in triplicate [66].

The half-maximal inhibitory concentration (IC_50_) values were calculated to evaluate the potency of compounds **5a**–**c** by fitting the investigational data with a 4-parameter logistic curve to obtain the dose-effect curves. The experimental IC_50_ results are expressed as mean values ± standard deviation (SD) of the three biological replicates.

Two-way analysis of variance (ANOVA) with a post hoc Bonferroni test was used to statistically examine the relative cell viability (%), compared to the negative control (NC), which was 100%. Data were analyzed using the PRISM 5 software (GraphPad Prism 8.0.2. Software, La Jolla, CA, USA). In terms of statistical analysis, the results were considered different at values of *p* < 0.05 or *p* < 0.001.

The selectivity index (SI) was used as a parameter to determine how many times more cytotoxic the investigated compounds were by exposing them to normal and cancer cells. It was calculated using the IC_50_ value ratio between the BJ cell line and the HepG2 cell line, both obtained from ATCC (Manassas, VA, USA). Results that are greater than 2 are considered suitable, as reported in the literature [30].

### 3.7. ADME Studies

#### 3.7.1. In Silico

An in silico molecular simulation technique was employed in the drug design of compounds **5a**–**c**, aiming to identify which compounds comply with several rules and properties to become drug candidates. The physicochemical and pharmacokinetic (PK) profiles of compounds **5a**–**c** were predicted using the ADMETlab 3.0 online platform [50]. The desired compounds were converted into SMILES format and introduced into the platform for the simultaneous prediction of all three compounds.

The considered physicochemical descriptors included the molecular weight (MW, g/mol), number of rotatable bonds (No. RB), number of hydrogen bond acceptors (No. HA), number of hydrogen bond donors (No. HD), topological polar surface area (TPSA, Å), octanol–water partition coefficient (logP), logarithm of aqueous solubility (logS), and the number of violations of Lipinski’s rule of five. These are key properties involved in assessing the drug-likeness of the desired compounds [2,19,27,28].

The selected pharmacokinetic descriptors included Caco-2 permeability and P-glycoprotein (P-gp) substrate probability for absorption; blood–brain barrier (BBB) permeability and plasma protein binding (PPB, %) for distribution; the likelihood of cytochrome P450 (CYP) 2C19, CYP2C9, CYP2D6, and CYP3A4 inhibition as well as human liver microsomal (HLM) stability for metabolism; and plasma clearance (Cl plasma, mL/min/kg) and half-life (T_1/2_, hours) for elimination [19].

#### 3.7.2. In Vitro Lipophilicity

The lipophilic properties of the three quinazoline-4(3*H*)-one derivatives (**5a**–**c**) were determined using an experimental assay and theoretical methods. The reagents and solvents utilized were purchased from authorized distributors [49].

The chromatographic performance of compounds **5a**–**c** was investigated by reverse-phase thin-layer chromatography (RP-TLC). Standardized Silica gel 60 RP-18 F_254_S chromatographic plates (20 × 10 cm, Merck, Darmstadt, Germany) were used as the stationary phase, and an *i*-propanol–water mixture, in volumetric proportions ranging from 40% to 60% (*v*/*v*) *i*-propanol, was used as the mobile phase. The variance between two successive elution mixtures was 5%.

Solutions containing 1 mg/mL of compounds **5a**–**c** were prepared in dimethylsulfoxide (DMSO). A volume equal to 2 μL of each sample was manually applied using chromatographic capillary micropipettes, at a distance of 10 mm from the lower side of the plates and 10 mm from their lateral sides. The gap between two successive spots was 10 mm.

The development process was carried out at room temperature using a chromatographic chamber pre-saturated for 30 min with mobile-phase vapors. The migration distance of the mobile phase was 80 mm for all concentrations used, and it was determined manually by calculating the retention factor R_f_; thus, R_f_ = migration distance of the compound/migration distance of the mobile phase. R_f_ values were calculated for each tested compound at all five concentrations of the mobile phase.

The spots were visualized under UV light at a wavelength of 254 nm.

Starting from the R_f_ values determined by RP-TLC, a series of lipophilicity parameters was calculated. Therefore, based on the Bate-Smith and Westall equation, the logarithmic R_M_ values were obtained [67]:

R_M_ = log [1/R_f_−1] (2)

Based on the linear relationship between the R_M_ value and the concentration of the organic solvent in the mobile phase [c], using the linear regression equation and the Soczewinski–Wachtmeister model (where b = slope of the line and c = concentration of organic modifier), the values of another lipophilicity parameter, R_M0_, were determined by extrapolating the R_M_ values to 0% organic modifier [68]:

R_M_ = R_M0_ + b[c] (3)

The arithmetic mean of the lipophilicity parameter R_M_ values, corresponding to a series of concentrations of the organic modifier in the mobile phase, was determined. Therefore, the mR_M_ lipophilicity parameter was obtained.

Applying principal component analysis (PCA, XL-STAT) to the lipophilicity parameter R_M_, new lipophilicity parameters represented by the principal components (PCs) were obtained [69,70].

Starting from the experimentally determined R_f_ values, the R_M_ logarithmic values were calculated. Consequently, the presence of minor errors in determining the R_f_ values can generate errors in calculating the R_M_ values. By analyzing the principal components, the errors in estimating the R_M0_ values are eliminated through extrapolation, thereby providing more precise results.

The chromatographic behavior of compounds **5a**–**c** was also examined by high-performance liquid chromatography coupled with mass spectrometry (HPLC-MS). The analyses were conducted using an Agilent 1100 series HPLC system (binary pump, autosampler, thermostat, UV detector, Agilent Technologies, USA) coupled to a Bruker Ion Trap SL system (Bruker Daltonics GmbH). Chromatographic separation was completed using a Zorbax SB-C18 column (50 mm × 2.1 mm i.d., 3.5 μm, Agilent Technologies). The mobile phase consisted of a mixture of water with 0.1% (*v*/*v*) acetic acid and acetonitrile. The elution gradient started at 15% acetonitrile and increased to 95% within 5 min. The flow rate was 0.6 mL/min, and the thermostat was set at 45 °C. UV detection was performed at 242 nm. Based on the retention times obtained, the capacity factor k was calculated using formula 3, where t_R_ = retention time of the compound and t_0_ = retention time of the solvent:

k = (t_R_ − t_0_)/t_0_, (4)

The logarithm of the capacity factor (log k) was used to estimate the lipophilicity of the compounds [71]. The RP-HPLC method has multiple advantages, including low sample requirements, good accuracy of determinations, fast analysis, and high reproducibility [72].

In order to interpret the obtained results, data were correlated with the theoretical logP values, calculated using the ADMETlab 3.0 online platform [50].

## 4. Conclusions

After a preliminary molecular docking analysis, three 2,3-disubstituted quinazolin-4(3*H*)-one derivatives (**5a**–**c**) were synthesized, starting with anthranilic acid via the nucleophilic addition of isothiocyanate derivatives (**2a**–**c**) in alkaline conditions, followed by a cyclization reaction; this yielded intermediates (**3a**–**c**), which were then involved in a nucleophilic *S*-alkylation reaction with 5-(2-bromoacetyl)-2-hydroxybenzamide in an alkaline environment.

Using their recorded IR, MS, ^1^H-NMR, and ^13^C-NMR spectral data, structural assignments of these compounds were carried out and confirmed according to the expected values.

By analyzing the preliminary molecular docking study, it was observed that compound **5b** had the highest binding affinity for VEGFR-2 (ΔG = −9.3 kcal/mol when using AutoDock Vina, and ΔG = −10.19 kcal/mol when using AutoDock). All compounds showed lower binding affinities for VEGFR-2 compared to sorafenib.

Regarding the study of molecular dynamics, it was found that compounds **5a**–**c** did not destabilize the receptor and could stably bind to it. They formed fewer hydrogen bonds than sorafenib, but exhibited greater mobility compared to it. Compound **5b** showed the most promising interaction profile, due to the combination of low RMSD values and moderate hydrogen bonds, although its molecular structure needs to be further optimized to increase the number of bonds and improve their stability and affinity.

Through the MM-PBSA study, using different substituents in position 3 of the quinazolinone heterocycle in the structure of compounds **5a**–**c**, a clear difference was observed after the decomposition of the interaction energy with nearby amino acids, especially for the unwanted repulsive interactions generated at the active site of VEGFR-2, compared to sorafenib.

Through the DFT study, HOMO was found in the quinazolinone heterocycle region of compounds **5a**–**c**, while LUMO was found in the salicylamide fragment region of them. Correspondingly, the distribution of electrons in the molecular structure was similar in compounds **5a**–**c**, with electron-rich regions appearing near the oxygen atoms, which could be involved in nucleophilic interactions or hydrogen acceptor bonds, while electron-deficient regions appeared around the amide hydrogen atoms, with the potential to generate an electrophilic attack or to form hydrogen donor bonds.

Compounds **5a**–**c** were evaluated for their cytotoxic effects on one normal fibroblast cell line (BJ) and against one cancer cell line, human hepatocellular carcinoma (HepG2), after 48 h of cell exposure. Sorafenib was utilized as a reference agent. The results were expressed through IC_50_ (μM), relative cell viability (%), and selectivity index values. The results of the in vitro assay suggested that **5a** was less cytotoxic against BJ cells than sorafenib and the other compounds. Compound **5b** displayed the most promising antitumor potential against the HepG2 cell line, being the most cytotoxic, but not in the same manner as sorafenib. Compound **5c** exhibited higher cytotoxic effects against BJ cells than compound **5a**, but lower than those of compound **5b** and sorafenib. Against HepG2 cells, **5c** displayed the weakest cytotoxic potential compared to the other tested compounds and the reference agent.

An in silico ADME study was performed on **5a**–**c** compounds using the ADMETlab 3.0 web tool, one of the latest available online prediction platforms. The compounds were characterized through physicochemical and pharmacokinetic descriptors. Based on the results, the screened compounds exhibited good druggability properties, with no violations of Lipinski’s rule of five. However, their predicted pharmacokinetic profile indicated low gastrointestinal absorption, potential to interfere with the metabolism of other drugs, and low clearance, with ultra-short or intermediate half-lives. Also, compound **5c** was predicted to have a higher stability when metabolized through HLM.

A variety of lipophilicity parameters (R_M_, mR_M_, R_M0_, PC_1,_ PC_2_, log k) were determined, derived from the retention factor (R_f_) obtained by the RP-TLC method, and the retention time (t_R_) obtained by the RP-HPLC method. The obtained results from both in vitro methods were consistent with the theoretical logP values, determined using an in silico method.

The most important functional groups noted were the oxygen atom in quinazolinone, the hydroxyl group in the salicylamide fragment, and the hydrophobic substituent, benzyl, found in position 3 of the quinazolinone heterocycle. The most potent compound in this study was **5b**. Further studies to optimize its molecular structure by increasing the number of interactions it can have with its target, VEGFR-2, while also modulating its druggability properties, could lead to the development of a HIT or LEAD candidate.

## Figures and Tables

**Table 1:** The binding affinities (ΔG) of compounds **5a**–**c** and **sorafenib** to the ATP-binding site from VEGFR-2 (kcal/mol).

Compounds	AutoDock Vina 1.1.2	AutoDock 4.2
**5a**	−8.40	−9.98
**5b**	−9.30	−10.19
**5c**	−8.20	−9.69
**Sorafenib**	−10.60	−11.97

**Table 2:** Summary of molecular dynamics analyses, including backbone RMSD of VEGFR-2 (RMSD-protein), RMSF of amino acid sidechains and alpha carbons, RMSD of ligand heavy atoms (RMSD-ligand), receptor radius of gyration (Rg), and average number of hydrogen bonds (NoHB) between ligand and VEGFR-2.

System Evaluated	RMSD—Protein (nm)	RMSF Amino Acid αCarbons (nm)	RMSF Amino Acid Sidechains (nm)	RMSD—Ligand (nm)	Rg Protein (nm)	NoHB Protein–Ligand (no/ns) and (SD)
**VEGFR-2 receptor + 5a**	0.26	0.12	0.17	0.30	2.09	1.05 (0.54)
**VEGFR-2 receptor + 5b**	0.19	0.11	0.16	0.33	2.08	1.37 (0.39)
**VEGFR-2 receptor + 5c**	0.22	0.11	0.16	0.28	2.07	1.04 (0.43)
**VEGFR-2 receptor + Sorafenib**	0.24	0.12	0.17	0.11	2.04	3.86 (0.13)
**VEGFR-2 receptor**	0.19	0.11	0.16	N/A	2.05	N/A

RMSD = root-mean-square deviation; RMSF = root-mean-square fluctuation; SD = standard deviation.

**Table 3:** The binding free energy of compounds **5a**–**c** and **sorafenib** to VEGFR-2 and their decomposition with different types of interaction (kcal/mol).

Compound	ΔG	Van Der Waals	Electrostatic	Solvation
**5a**	−32.29	−45.45	−17.35	30.51
**5b**	−35.33	−53.01	−7.32	25.00
**5c**	−32.64	−45.45	−14.81	27.62
**Sorafenib**	−69.39	−59.19	−39.67	29.47

**Table 4:** The energy levels of the frontier molecular orbitals (eV).

Compound	Vacuum		Nonpolar Solvent (ε = 7.43)		Polar Solvent (ε = 37.22)		Water	
	HOMO	LUMO	HOMO	LUMO	HOMO	LUMO	HOMO	LUMO
**5a**	−6.18	−1.82	−6.26	−1.76	−6.27	−1.75	−6.28	−1.75
**5b**	−6.19	−1.75	−6.24	−1.73	−6.26	−1.74	−6.26	−1.74
**5c**	−6.15	−1.83	−6.26	−1.83	−6.27	−1.82	−6.27	−1.82

**Table 5:** Some molecular descriptors important for compounds **5a**–**c**.

Compound	Area (Å^2^)	Volume (Å^3^)	Dipole (D)
**5a**	394.48	375.02	1.44
**5b**	438.03	425.95	1.35
**5c**	377.65	360.31	2.60

**Table 6:** Calculated IC_50_ values (μM) at 48 h after exposure of the human normal foreskin fibroblasts (BJ) and human hepatocellular carcinoma (HepG2) to **5a**–**c** and sorafenib, and the selectivity indexes (SI).

Co		IC_50_ (μM) at 48 h		Selectivity Index (SI)
	Tested Concentration (μM)	Cells Line		
		BJ	HepG2	48 h
**5a**	12.50–150.00	>100.00	>50.00	-
**5b**	12.50–150.00	26.60 ± 1.07	24.87 ± 0.19	1.06
**5c**	12.50–150.00	34.50 ± 1.09	25.46 ± 0.20	1.35
**Sorafenib**	0.30–7.50	5.11 ± 0.16	4.87 ± 0.19	1.04

Co = compounds; IC_50_ values are represented as the average of three values ± standard deviation (SD); SI = IC_50_ BJ/IC_50_ HepG2.

**Table 7:** The computed in silico physicochemical descriptors for compounds **5a**–**c.**

Co	MW (g/mol)	No. RB	No. HA	No. HD	TPSA (Å)	logP	logS	No. of Lipinski Violations
**5a**	395.43	7	7	3	115.28	2.654	−4.313	0
**5b**	449.45	7	7	3	115.28	3.140	−4.231	0
**5c**	383.09	6	7	3	115.28	2.419	−4.036	0

Co = compounds; MW = molecular weight; No. RB = number of rotatable bonds; No. HA = number of hydrogen bond receptors; No. HD = number of hydrogen bond donors; TPSA = topological polar surface area; logP = octanol–water partition coefficient; logS = logarithm of aqueous solubility.

**Table 8:** The computed in silico pharmacokinetic descriptors for compounds **5a**–**c**.

Co	Caco-2 Perm.	P-gp sb.	PPB (%)	CYP2C19 Inhibition	CYP2D6 Inhibition	CYP3A4 Inhibition	HLM Stability	Cl Plasma	T_1/2_
**5a**	395.43	No	97.9	Yes	No	Yes	Unstable	4.547	0.529
**5b**	449.45	No	98.7	Yes	No	Yes	Unstable	4.109	0.474
**5c**	383.09	No	98.1	Yes	No	Yes	Stable	4.889	0.526

Co = compounds; Perm. = permeability; P-gp sb. = P-glycoprotein substrate; PPB = plasma protein binding; HLM = human liver microsomal; Cl = plasma clearance; T_1/2_ = half-life.

**Table 9:** The values of the retention parameters R_f_ and R_M_ for compounds **5a**–**c**.

Co	Isopropanol–Water (*v*:*v* %)									
	40:60		45:60		50:50		55:45		60:40	
	R_f_	R_M_	R_f_	R_M_	R_f_	R_M_	R_f_	R_M_	R_f_	R_M_
**5a**	0.43	0.12	0.48	0.03	0.55	−0.08	0.66	−0.28	0.8	−0.60
**5b**	0.30	0.36	0.37	0.23	0.46	0.06	0.58	−0.14	0.72	−0.41
**5c**	0.47	0.05	0.51	−0.01	0.58	−0.14	0.68	−0.32	0.81	−0.62

Co = compound; R_f_ = retention factor − the migration distance of the compound/the migration distance of the mobile phase; R_M_ = the logarithmic values of R_f_.

**Table 10:** The values of mR_M_, R_M0_, b, R^2,^ and log k for compounds **5a**–**c**.

Co	RP-TLC				RP-HPLC
	mR_M_	R_M0_	b	R^2^	log k
**5a**	−0.16	−0.03	1.60	0.93	0.93
**5b**	0.02	−0.03	1.95	0.98	0.99
**5c**	−0.21	−0.03	1.46	0.93	0.91

Co = compound; mR_M_ = the arithmetic mean of the lipophilicity parameter values R_M_; R_M0_ = extrapolation of the R_M_ values to the 0% organic modifier; b = the slope of the line; R^2^ = the coefficient of determination; log k = the value of the logarithm of the capacity factor.

**Table 11:** The scores obtained for the first two principal components.

Co	PC_1_	PC_2_
**5a**	0.64	−0.09
**5b**	−2.01	0.41
**5c**	1.37	−0.31

Co = compounds; PC_1_ = principal component 1; PC_2_ = principal component 2.

**Table 12:** Correlation coefficient values (R^2^) between experimental (mR_M_, R_M0_, PC_1_, log k) and theoretical values of lipophilicity parameter (logP) for compounds **5a**–**c**.

Lipophilicity Parameter	mR_M_	R_M0_	PC_1_	log k	logP
**mR_M_**	1.00	−0.97	−0.99	0.99	0.99
**R_M0_**	−0.97	1.00	0.97	−0.98	0.99
**PC_1_**	−0.99	0.97	1.00	−0.99	0.99
**log k**	0.99	−0.98	−0.99	1.00	0.99
**logP**	0.99	−0.99	−0.99	0.99	1.00

mR_M_ = the arithmetic mean of the lipophilicity parameter values R_M_; R_M0_ = extrapolation of R_M_ values to 0% organic modifier; PC_1_ = principal component 1; log k = the value of the logarithm of the capacity factor; logP = octanol–water partition coefficient.

## Data Availability

Data is contained within the article or Supplementary Materials.

## Supplementary Materials

The following supporting information can be downloaded at: https://www.mdpi.com/article/10.3390/molecules30244719/s1, Figure S1: The IR spectrum for compound **5a**; Figure S2: The IR spectrum for compound **5b**; Figure S3: The IR spectrum for compound **5c**; Figure S4: The MS spectrum for compound **5a**; Figure S5: The MS spectrum for compound **5b**; Figure S6: The MS spectrum for compound **5c**; Figure S7: The ^1^HNMR spectrum for compound **5a**; Figure S8: The ^1^HNMR spectrum for compound **5b**; Figure S9: The ^1^HNMR spectrum for compound **5c**; Figure S10: The ^13^CNMR spectrum for compound **5a**; Figure S11: The ^13^CNMR spectrum for compound **5b**; Figure S12: The ^13^CNMR spectrum for compound **5c**. Figure S13: The top superposing binding conformation of compound **5a** and **sorafenib** at the ATP-binding site of VEGFR-2 (carbon atoms in magenta for compound **5a** and in black for **sorafenib**, oxygen atoms in red, hydrogen atoms in white, nitrogen atoms in blue, sulfur atom in yellow). Figure S14: The top superposing binding conformation of compound **5c** and **sorafenib** at the ATP-binding site of VEGFR-2 (carbon atoms in magenta for compound **5c** and in black for **sorafenib**, oxygen atoms in red, hydrogen atoms in white, nitrogen atoms in blue, sulfur atom in yellow). Figure S15: The conformation that represents the highest occupied molecular orbital (HOMO) of compound **5a**. Figure S16: The conformation that represents the lowest unoccupied molecular orbital (LUMO) of compound **5a**. Figure S17: The conformation that represents the highest occupied molecular orbital (HOMO) of compound **5b**. Figure S18: The conformation that represents the lowest unoccupied molecular orbital (LUMO) of compound **5b**. Figure S19: The conformation that represents the highest occupied molecular orbital (HOMO) of compound **5c**. Figure S20: The conformation that represents the lowest unoccupied molecular orbital (LUMO) of compound **5c**. Figure S21: A molecular electrostatic potential (MEP) map of compound **5a**. Figure S22: A molecular electrostatic potential (MEP) map of compound **5b**. Figure S23: A molecular electrostatic potential (MEP) map of compound **5c**. Table S1. The number of hydrogen bonds between the studied compounds **5a**–**c**, **sorafenib**, and VEGFR-2 calculated every 10 ns.
