# Supplementary material for: New Quinazolin-4(3H)-one Derivatives as Potential Antitumoral Compounds: Synthesis, In Vitro Cytotoxicity Against the HepG2 Cell Line, and In Silico VEGFR-2 Targeting-Based Studies
Source: Molecules. 2025 Dec 9;30(24):4719. doi: 10.3390/molecules30244719 (PMC12736141; doi:10.3390/molecules30244719)
Supplement: Supplementary file 1 [file molecules-30-04719-s001.zip › molecules-3989074-supplementary.pdf]

molecules

MDPI

Supplementary Materials

1. Figures

1.1. The IR Spectra

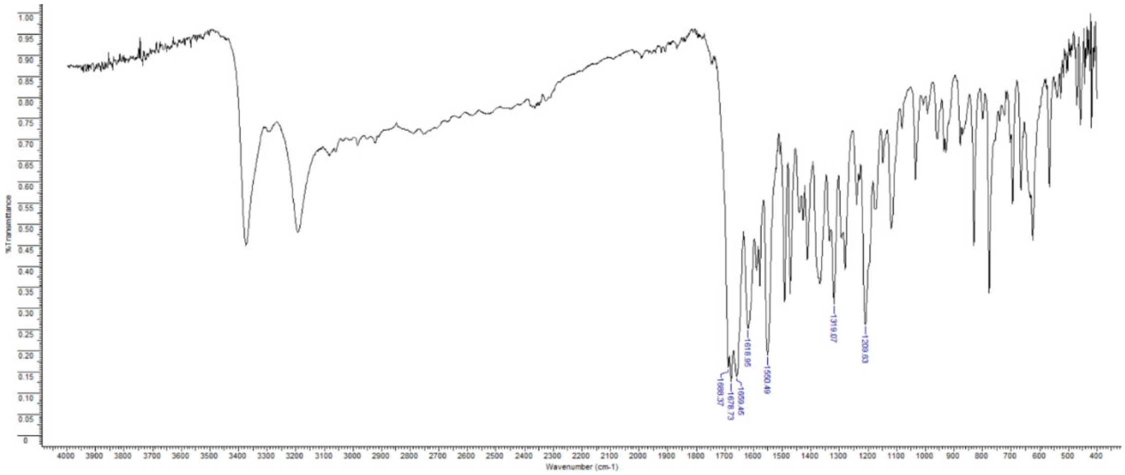

Figure S1. The IR spectrum for compound **5a**.

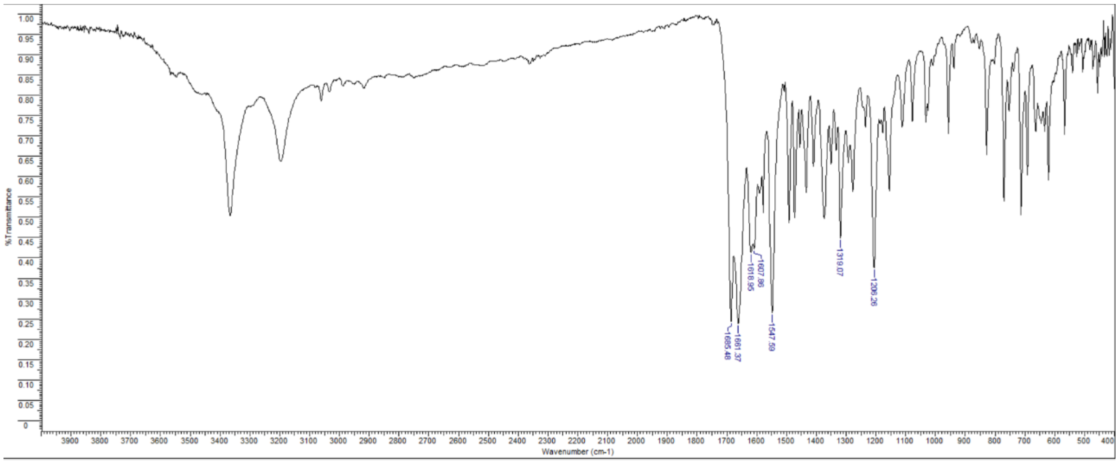

Figure S2. The IR spectrum for compound **5b**.

 https://doi.org/10.3390/molecules30244719

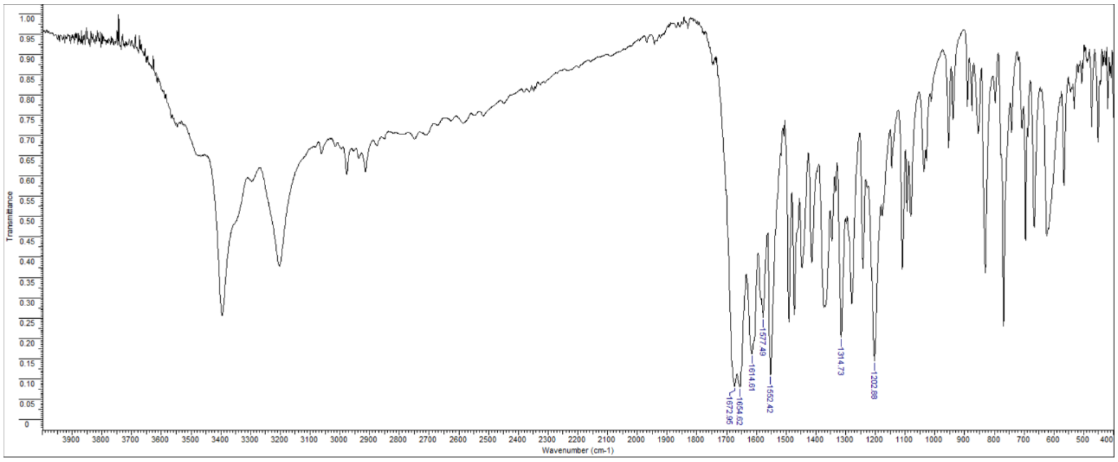

Figure S3. The IR spectrum for compound **5c**.

1.2. The MS Spectra

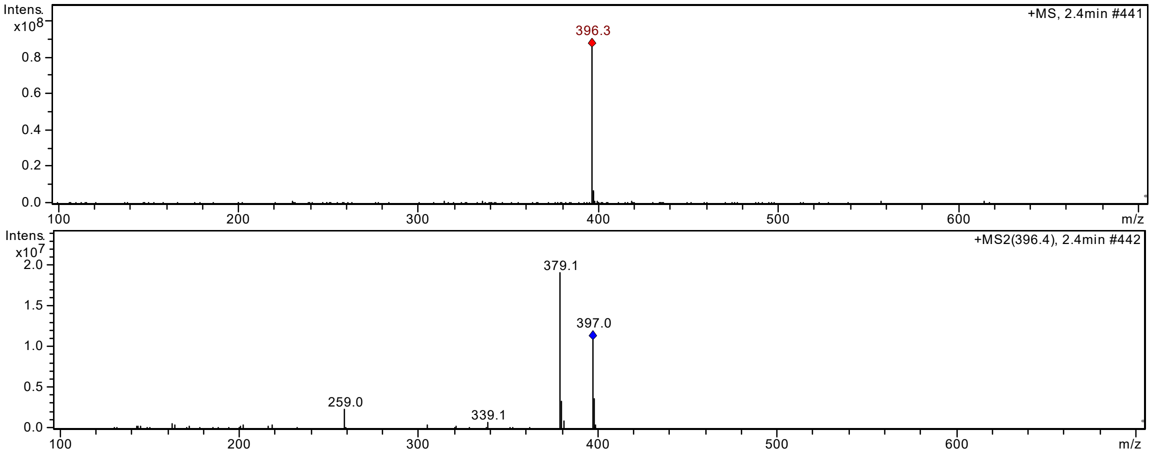

Figure S4. The MS spectrum for compound **5a**.

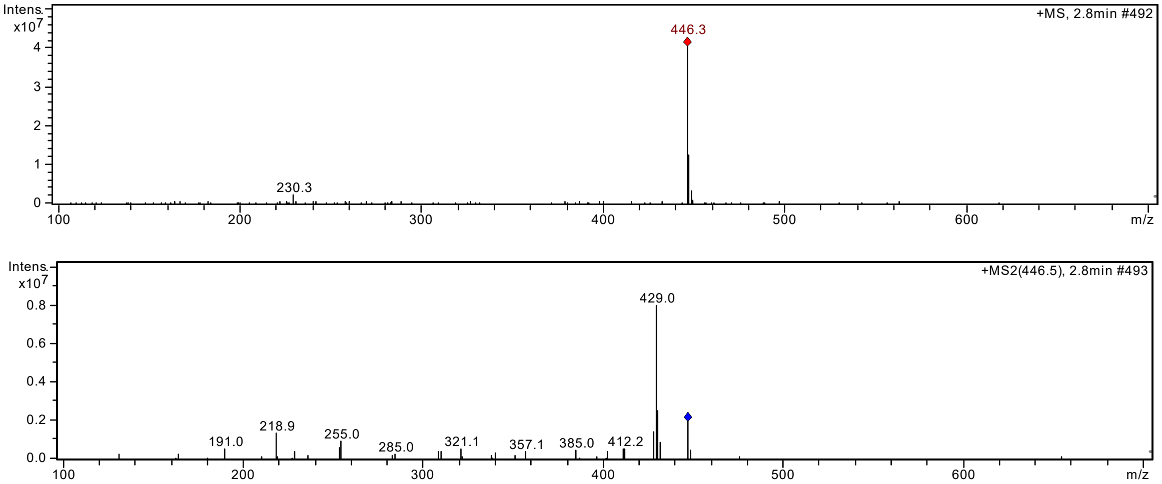

Figure S5. The MS spectrum for compound **5b**.

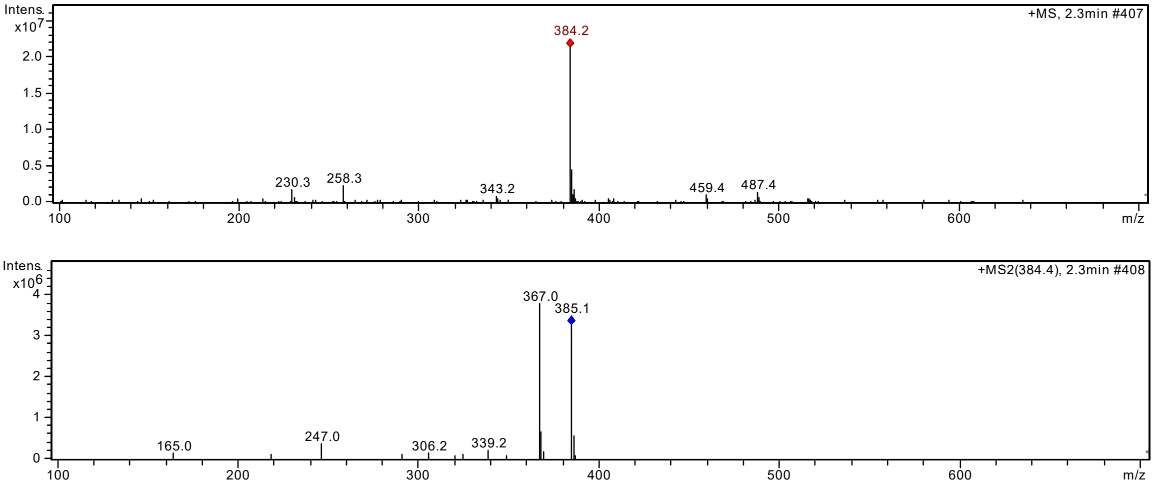

Figure S6. The MS spectrum for compound **5c**.

1.3. ¹HNMR Spectra

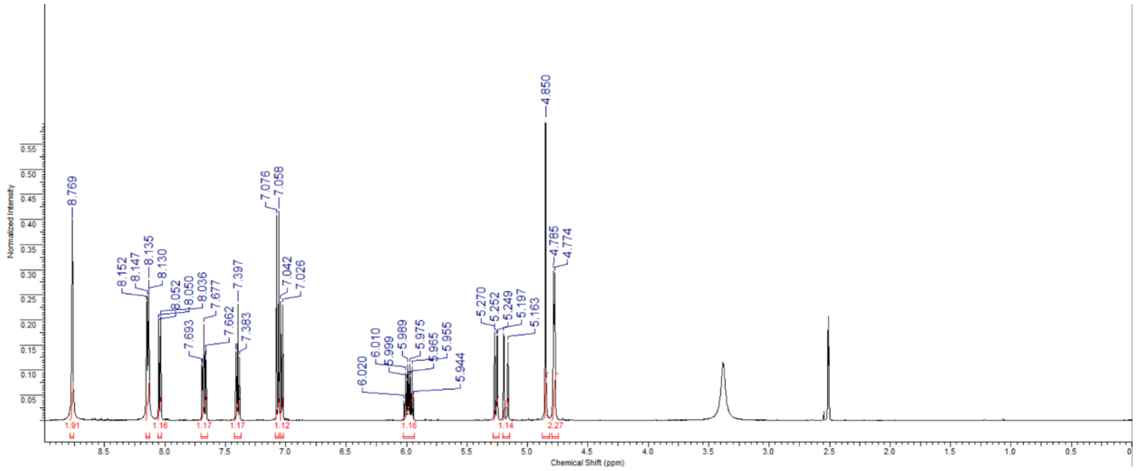

Figure S7. The ¹HNMR spectrum for compound **5a**.

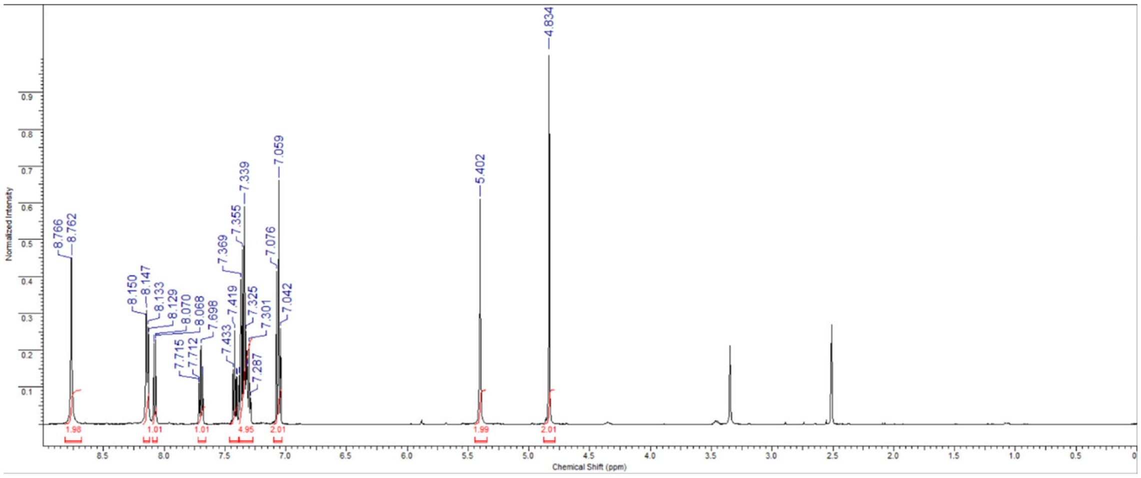

Figure S8. The ¹HNMR spectrum for compound **5b**.

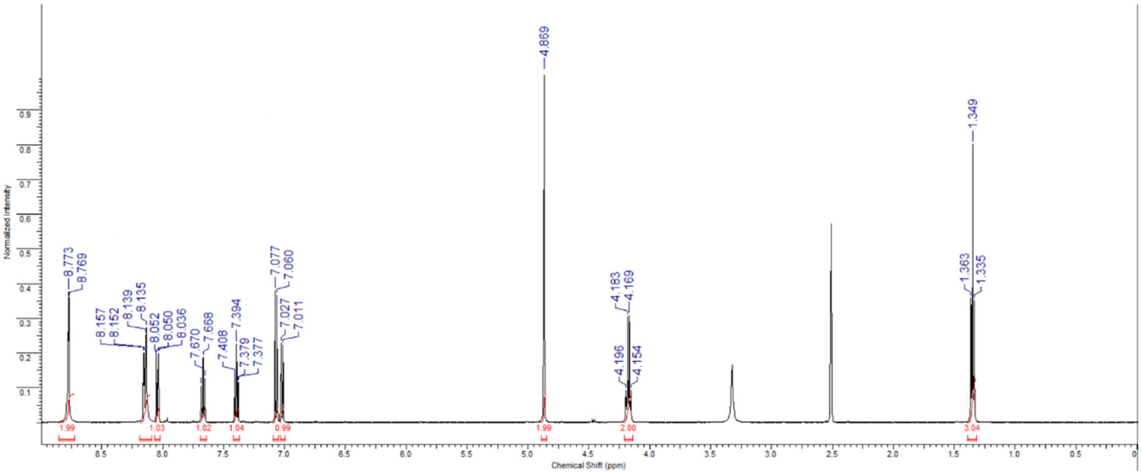

Figure S9. The ^{1}HNMR spectrum for compound **5c**.

1.4. ^{13}CNMR Spectra

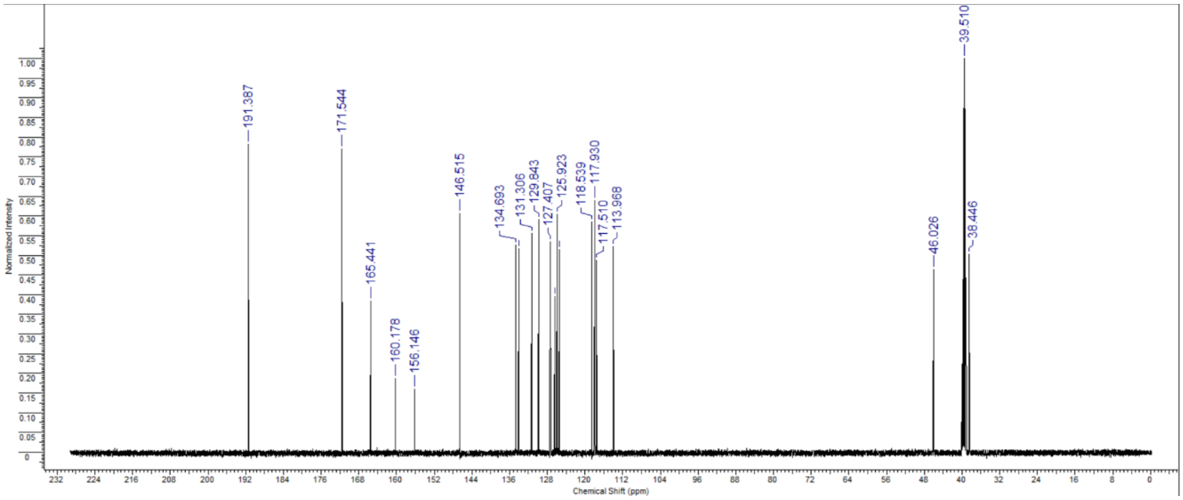

Figure S10. The ^{13}CNMR spectrum for compound **5a**.

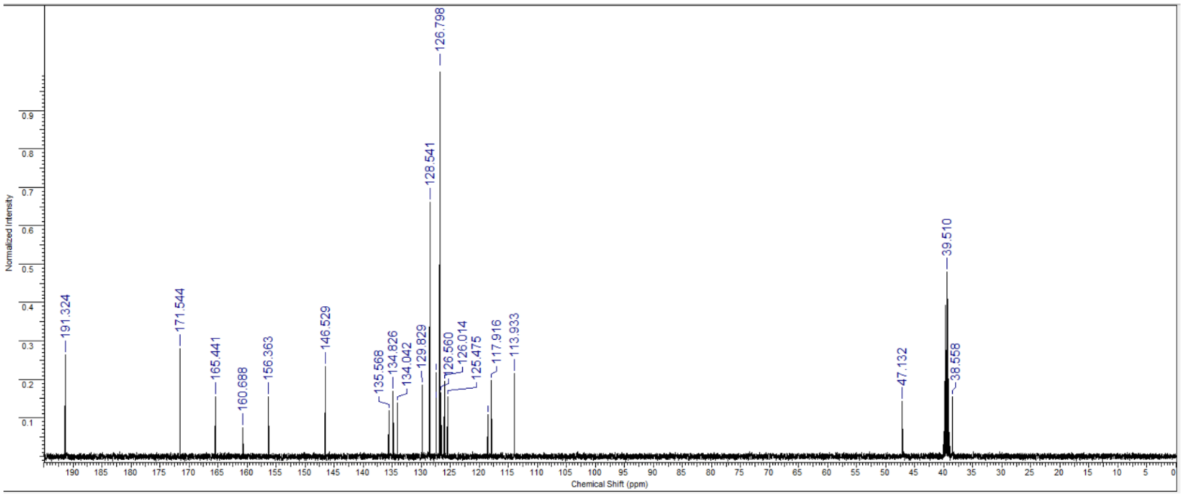

Figure S11. The ^{13}CNMR spectrum for compound **5b**.

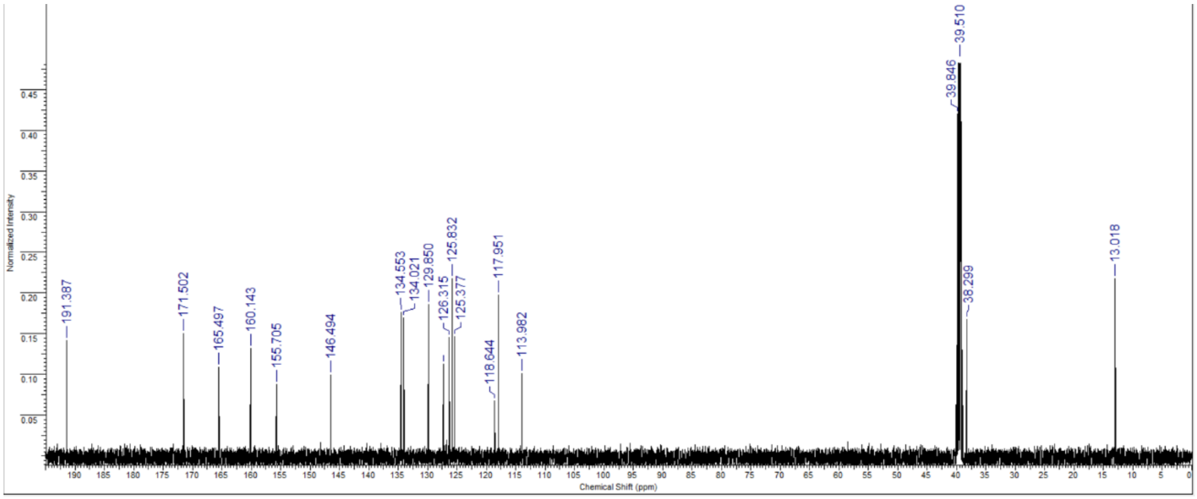

Figure S12. The ^{13}CNMR spectrum for compound **5c**.

1.5. Molecular Docking

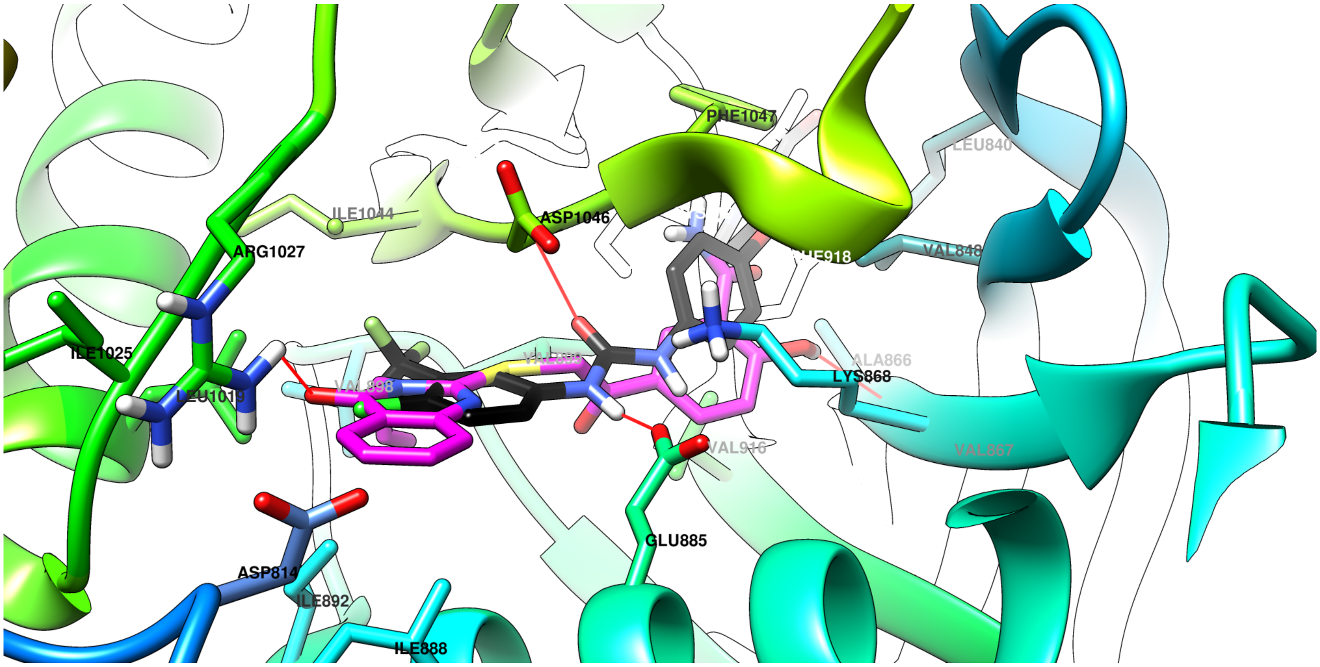

Figure S13. The top superposing binding conformation of compound **5a** and **sorafenib** at the ATP-binding site of VEGFR-2 (carbon atoms in magenta for compound **5a** and in black for **sorafenib**, oxygen atoms in red, hydrogen atoms in white, nitrogen atoms in blue, sulphur atom in yellow).

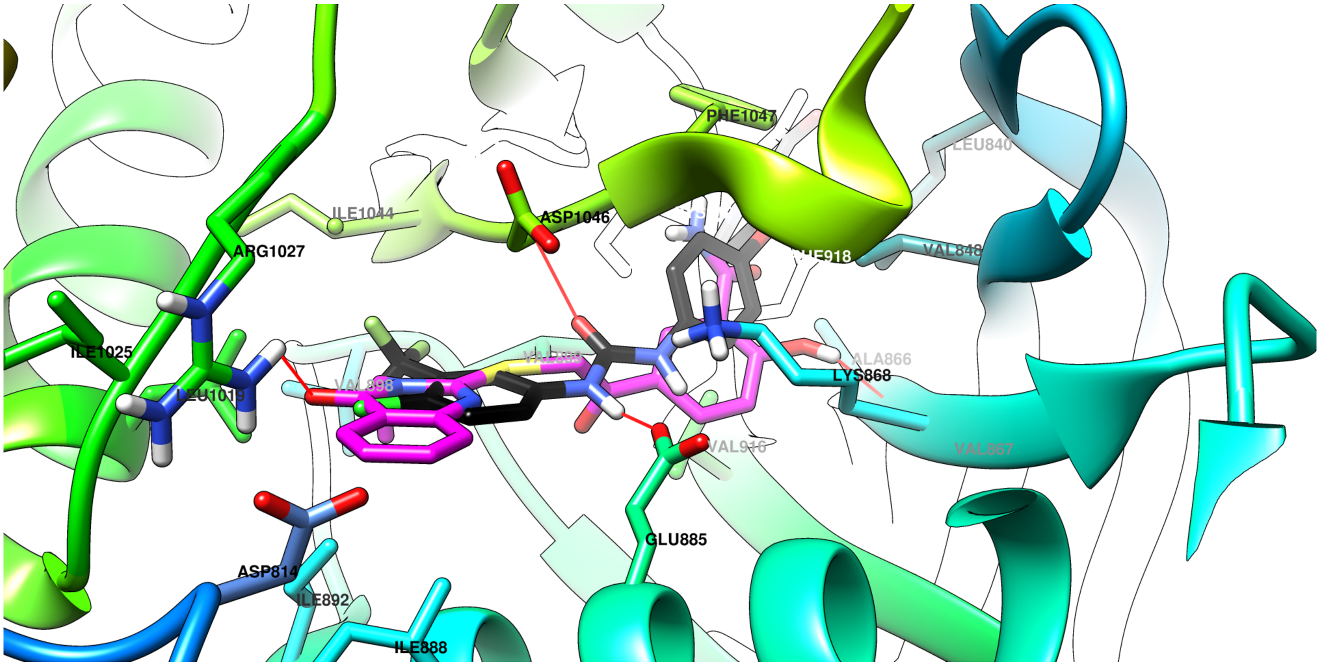

Figure S14. The top superposing binding conformation of compound **5c** and **sorafenib** at the ATP-binding site of VEGFR-2 (carbon atoms in magenta for compound **5c** and in black for **sorafenib**, oxygen atoms in red, hydrogen atoms in white, nitrogen atoms in blue, sulphur atom in yellow).

1.6. Density Functional Theory (DFT)

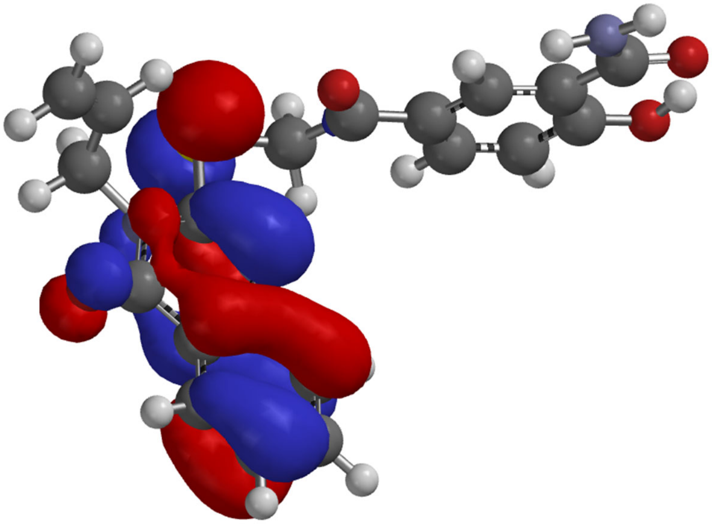

Figure S15. The conformation that represents the highest occupied molecular orbital (HOMO) of compound **5a**.

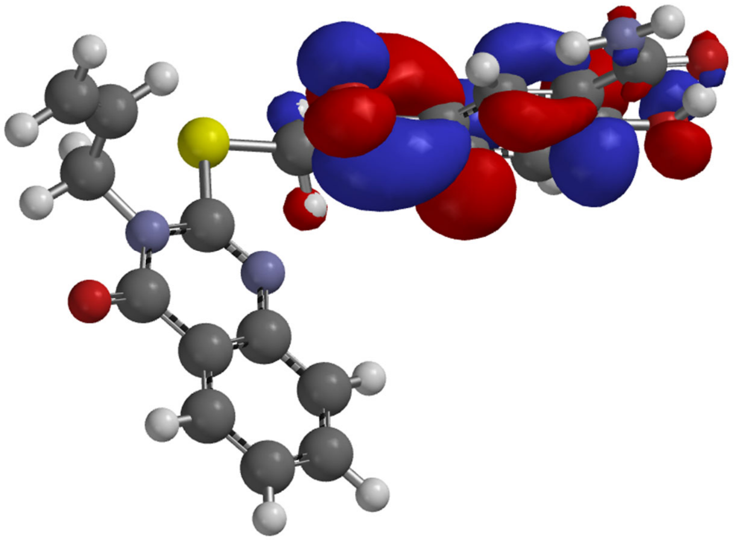

Figure S16. The conformation that represents the lowest unoccupied molecular orbital (LUMO) of compound **5a**.

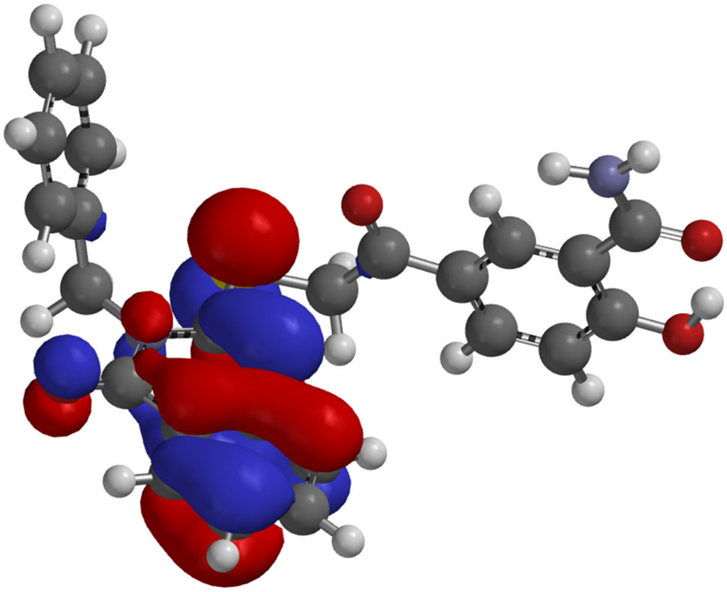

Figure S17. The conformation that represents the highest occupied molecular orbital (HOMO) of compound **5b**.

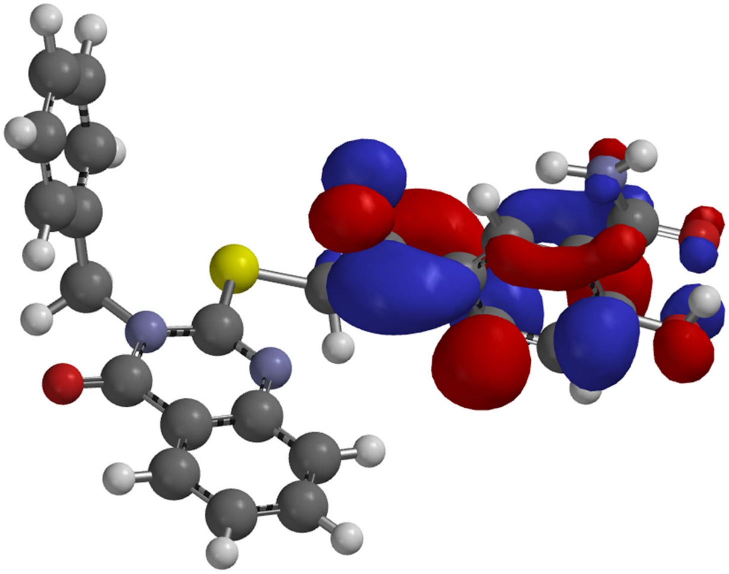

Figure S18. The conformation that represents the lowest unoccupied molecular orbital (LUMO) of compound **5b**.

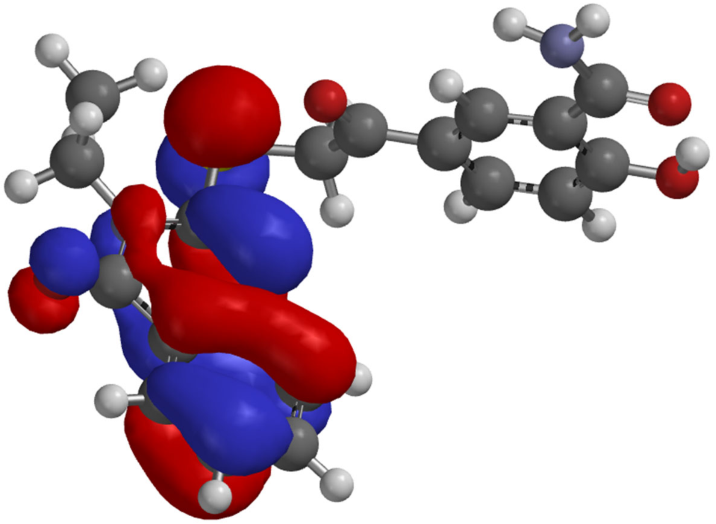

Figure S19. The conformation that represents the highest occupied molecular orbital (HOMO) of compound **5c**.

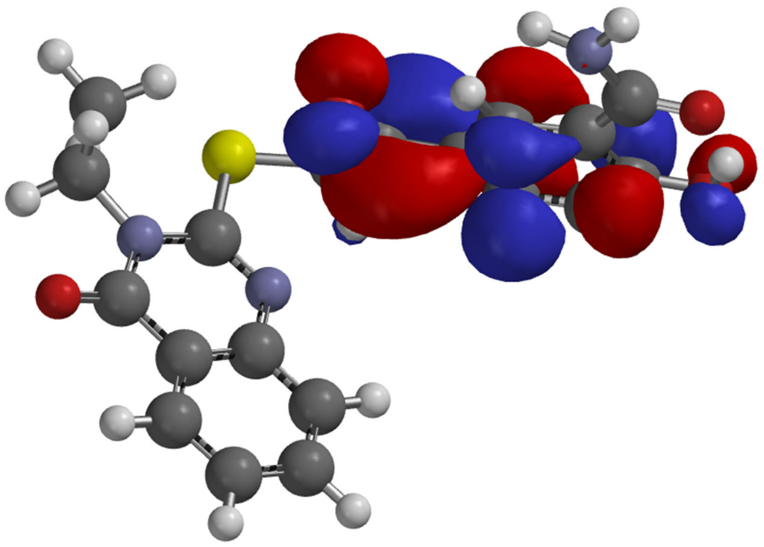

Figure S20. The conformation that represents the lowest occupied molecular orbital (LUMO) of compound **5c**.

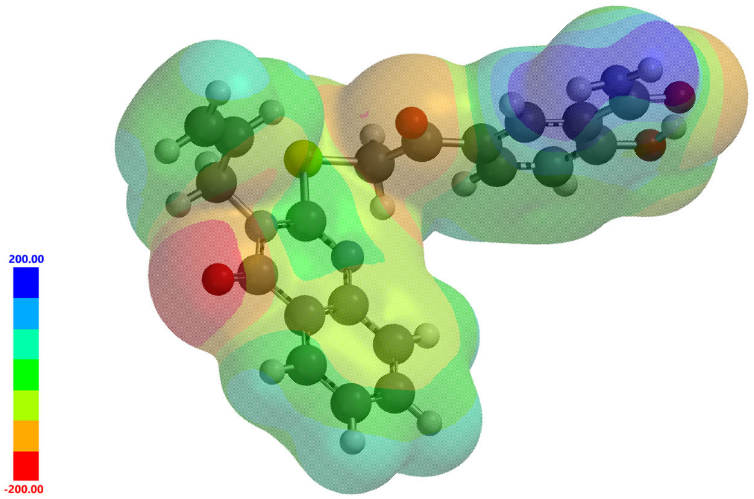

Figure S21. A molecular electrostatic potential (MEP) map of compound **5a**.

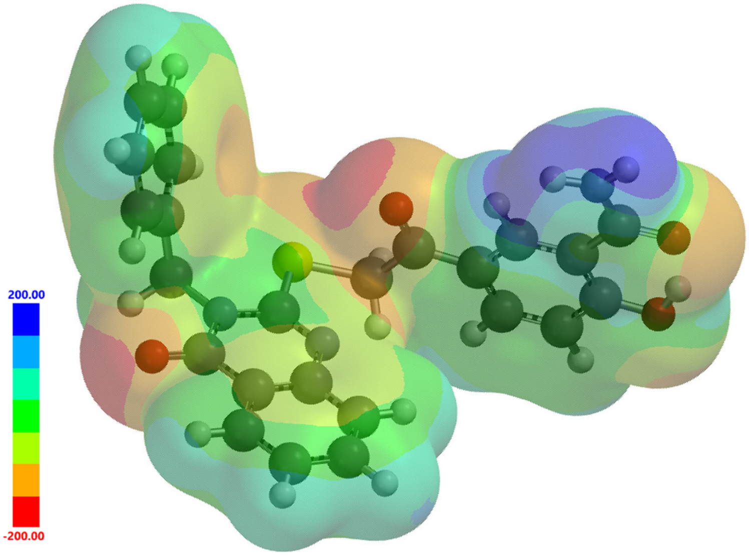

Figure S22. A molecular electrostatic potential (MEP) map of compound **5b**.

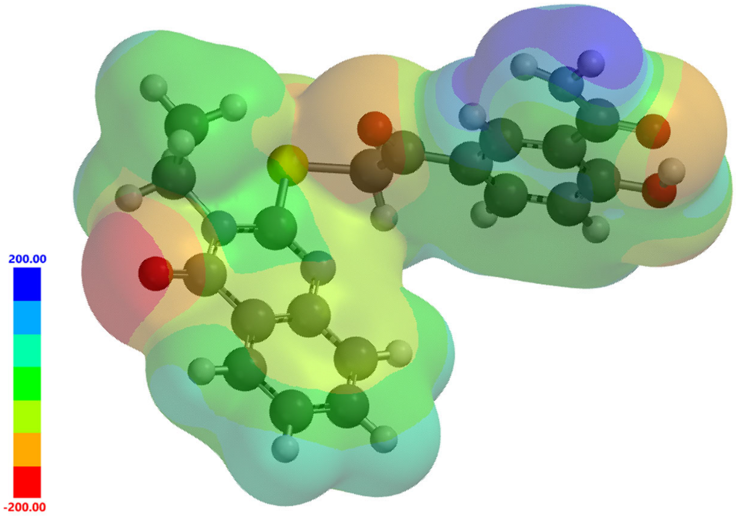

Figure S23. A molecular electrostatic potential (MEP) map of compound **5c**.

2. Tables

Molecular Dynamics

Table S1. The number of hydrogen bonds between the studied compounds **5a-c**, **sorafenib**, and VEGFR-2 calculated every 10 ns.

Time (ns)	Compound 5a	Compound 5b	Compound 5c	Sorafenib
10	1	2	1	4
20	0	2	1	4
30	0	2	0	4
40	0	2	0	3
50	0	1	1	4
60	1	1	1	4
70	1	1	1	4

80	1	2	1	4
90	1	1	1	4
100	1	2	1	4
110	1	2	1	3
120	1	1	1	4
130	1	1	3	4
140	1	1	2	4
150	2	2	2	4
160	1	1	1	4
170	2	1	0	4
180	2	1	0	4
190	2	2	1	4
200	0	1	2	4
